# Sexual antagonism, mating systems, and recombination suppression on sex chromosomes

**DOI:** 10.1093/evlett/qrag021

**Published:** 2026-05-18

**Authors:** Ewan Flintham, Charles Mullon

**Affiliations:** Department of Ecology and Evolution, University of Lausanne, Lausanne, Switzerland; Department of Ecology and Evolution, University of Lausanne, Lausanne, Switzerland

**Keywords:** Recombination, Sex chromosomes, Sexual conflict, Polygyny, Genetic Polymorphism, Inversions

## Abstract

The suppression of recombination between sex chromosomes is a widespread feature of genetic sex determination systems. Competing explanations for this evolution fall into two broad categories: recombination arrest driven by the capture of sexually antagonistic variation vs. arrest driven without sex-specific selection. Using population genetic models, we compare the substitution rates of full recombination suppressors (such as inversions) driven by sexually antagonistic selection to those expected under neutrality. We consider both XY and ZW systems, mating ecologies ranging from random mating to polygyny with high male reproductive variance, suppressors arising on heterogametic (Y or W) vs. homogametic (X or Z) chromosomes, and the effects of deleterious alleles segregating at other loci on the chromosome. We find that even very weak sexual antagonism is sufficient to expedite the substitution of suppressors by orders of magnitude relative to neutrality. This acceleration is robust to the presence of deleterious variation, although high variance in male reproductive success can diminish substitution rates of Y-linked suppressors in XY systems. By contrast, in ZW systems, elevated male reproductive variance tends to favor faster recombination arrest via W-linked suppressors, leading to higher overall rates of suppression than in comparable XY systems. Despite stronger selection on Y/W-linked suppressors, we find that recombination arrest driven by sexual antagonism is often expected to arise from homogametic (X/Z-linked) suppressors, because their mutational input is higher and they can experience weaker drift. Together, these results yield testable predictions for the genomic distribution of sex-chromosome inversions across taxa differing in mating system and sex-determination system.

## Introduction

In many species with separate sexes, an individual’s sex is determined by its karyotype, in particular the identity of its sex chromosomes (Bachtrog et al., 2014). These chromosomes contain the gene or genes that instigate sex-specific development, which are held together in a genomic region that does not recombine in at least one sex (a sex-determining region, or “SDR,” Otto et al., 2011; Wright et al., 2016). For example, in XY species, sex is assigned according to the presence or absence of a dominant male-determining locus on the Y chromosome, around which recombination is suppressed in males. This situation is mirrored in ZW species, where a dominant female-determining factor sits on the W chromosome, with recombination suppression in females. Strong genetic linkage between sex-determining loci in the heterogametic sex is thought to be adaptive as it ensures alleles responsible for sexual development are co-inherited (Charlesworth & Charlesworth, 2005; Nei, 1969). However, regions of suppressed recombination that extend well beyond the vicinity of sex-determining loci—often encompassing the majority or even entirety of the chromosome—have evolved independently in many species (Bachtrog et al., 2014; Bergero & Charlesworth, 2009). Indeed, extensive recombination suppression in the heterogametic sex is considered a hallmark of a sex chromosome system (Otto, 2014).

The classical explanation for the evolution of recombination suppression on sex chromosomes is that selection favors tighter linkage between a sex-determining factor and alleles that are beneficial when expressed in that sex (i.e., that contribute to sex-specific adaptation, Beukeboom & Perrin, 2014; Fisher, 1931; Ponnikas et al., 2018; Wright et al., 2016). Theoretical population genetic models have been integral to our understanding of this process. Early models showed that selection favors the invasion of a Y-linked recombination suppressor if it captures a male SDR and the male-beneficial allele at a sexually antagonistic locus under balancing selection (modeled as a single di-allelic site with fixed fitness effects; Charlesworth & Charlesworth, 1980; Nei, 1969). Symmetrically, Charlesworth and Charlesworth (1980) also demonstrated that selection favors an X-linked suppressor if it captures the SDR homologue and the female-beneficial allele at such a locus. Lenormand (2003) later found that a modifier decreasing recombination by a small amount would be favored if it increased linkage between the SDR and a previously weakly linked polymorphic locus experiencing sex-specific selection—although without balancing selection, variation at such a locus would be transient, limiting the scope for selection on recombination. This result was then found to hold for most levels of linkage between a modifier, the SDR, and a locus under selection (Otto, 2014). Finally, Olito and Abbott (2025) and Olito et al. (2024) estimated the invasion probability of an inversion linking a Y-linked SDR with a di-allelic sexually antagonistic locus with weak fitness effects in a large, well-mixed population, and used this to make predictions about the chromosomal length affected by recombination suppression.

In contrast to the classic sexual antagonism explanation, a more recent group of models has shown that it is possible for recombination suppression on sex chromosomes to evolve without sex-specific selection (reviewed in Charlesworth & Olito, 2024; Jay et al., 2024; Olito & Abbott, 2025; Ponnikas et al., 2018). Here, recombination suppressors capture the SDR and a nearby region of the chromosome that is either (i) neutral (i.e., contains no segregating loci affecting fitness; Charlesworth & Charlesworth, 2005; Ironside, 2010; Jeffries et al., 2021) and then fixes by chance owing to genetic drift; or (ii) includes loci at which deleterious recessive variants segregate in the population. In this second case, a “lucky” suppressor mutant that happens to appear in a particularly unloaded background (i.e., captures a set of loci at which fewer than average deleterious alleles are present; Lenormand & Roze, 2022) experiences a transient fitness advantage that helps drive its fixation (Charlesworth & Olito, 2024; Jay et al., 2024; Olito & Abbott, 2025; Olito et al., 2024), although the fixation probabilities of these suppressors generally remain well below those of neutral alleles (Roze & Lenormand, 2025). In both scenarios, recombination arrest must later be stabilized in the face of mutations that restore recombination (e.g., reversions; Lenormand & Roze, 2024). This may occur through the development of structural incompatibilities between sex chromosome homologues after recombination arrest (Jay et al., 2024), or through sex-specific divergence in gene regulation at loci within the non-recombining region, leading to low-fitness recombinants (Lenormand & Roze, 2022).

While theory has demonstrated that sexual antagonism and the fixation of neutral or lucky suppressors can in principle drive recombination arrest, the relative contribution of these mechanisms to sex chromosome evolution, as well as the genetic signatures they may be associated with, remains understudied (Jay et al., 2024; Lenormand & Roze, 2022; Olito & Abbott, 2025; Ponnikas et al., 2018). In particular, it is not known how strongly sexual antagonism, genetic drift, and selection on deleterious variation affect the substitution of recombination suppressors, and whether they do so similarly for modifiers arising in different sex-linked regions. For example, sexually antagonistic polymorphisms generate stronger positive selection on full suppressors arising on heterogametic (Y or W) than on homogametic (X or Z) sex chromosomes (Blackmon & Brandvain, 2017; Charlesworth & Charlesworth, 1980), but the impact of this asymmetry on evolutionary rates—once mutation and drift are taken into account—remains underexplored. Meanwhile, studies considering lucky inversions have predominantly focused on fixation probabilities in autosomes and Y chromosomes (Charlesworth & Olito, 2024; Jay et al., 2024; Roze & Lenormand, 2025), whereas substitution rates on homogametic chromosomes are yet to be quantified. Consequently, we lack predictions for whether recombination evolution under different models should occur on comparable timescales, or involve modifiers with similar genomic distributions. Yet with the increasing availability of large cross-taxa genomic datasets (e.g., Jeffries et al., 2025), such predictions may prove useful in delineating the mechanisms driving sex chromosome evolution by providing tests for comparative studies.

Characterizing the tempo of recombination evolution on sex chromosomes, however, is not straightforward. The substitution rates of recombination modifiers depend on multiple factors, including the nature of segregating sexually antagonistic and deleterious variation, as well as the strength of genetic drift experienced by sex-linked genomic regions and on demographic parameters such as the mating system (Laporte & Charlesworth, 2002; Vicoso & Charlesworth, 2009). Indeed, by modulating the effective population sizes of sex-linked genomic regions, mating system variation may have especially strong effects on the relative rates of recombination evolution along different sex chromosomes (e.g., X vs. Y), as well as the pace of recombination suppression in different sex-determination systems (e.g., in XY vs. ZW systems). However, the consequences of these effects for suppressor substitution rates across X, Y, Z, and W chromosomes have yet to be characterized or integrated into existing theory on sex chromosome evolution.

Here, we address some of these issues by analyzing a series of mathematical and computational models of recombination suppression to better understand the tempo of sex chromosome evolution. We do this under different assumptions about the segregation of sexually antagonistic and universally deleterious alleles, as well as the mating and sex-determination systems. Specifically, we first characterize the nature of sexually antagonistic variation likely to evolve on the PAR. Starting with the Y chromosome as a basis, we then investigate the strength of selection for recombination suppression driven by sexual antagonism by calculating the fixation probability and substitution rate of a full recombination suppressor capturing a sex-specifically selected locus. We then extend our analyses to consider fixation probabilities for recombination suppressors appearing in different sex-linked regions (X, Y, Z, and W chromosomes) and under different mating systems. Finally, we use individual-based simulations to estimate fixation probabilities for recombination suppressors that can capture a sexually antagonistic locus at sites at which ubiquitously deleterious alleles segregate.

## Model

### Lifecycle and fitness

We consider a diploid population of large and constant size *N*, composed of equal numbers of adult males and females, with non-overlapping generations. Males and females reproduce to generate zygotes whose sex is genetically determined such that the primary sex ratio is unbiased. Adults die, and juveniles compete sex-specifically to become the male and female adults of the next generation. As a baseline, reproduction occurs through the random union of male and female gametes, but later we incorporate a more flexible mating model that allows for male-biased reproductive variance.

Each individual expresses a quantitative trait *z* that influences its reproductive success in a sex-specific manner. We denote male and female fecundity by $w_\mathrm{m}(z)$ and $w_\mathrm{f}(z)$, respectively. Unless otherwise stated, we assume that these are given by Gaussian functions: $w_{\mathrm{m}}(z) \propto e^{-\sigma _\mathrm{m}(1+z)^2}$ and $w_{\mathrm{f}}(z) \propto e^{-\sigma _\mathrm{f}(1-z)^2}$ (as in, e.g., Connallon & Clark, 2014a, b; Flintham, 2025; Lande, 1980; Muralidhar & Coop, 2024), such that male fitness is maximized by trait value $z=-1$ and female fitness by $z=1$, where $\sigma _\mathrm{m}$ and $\sigma _\mathrm{f}$ determine how steeply fitness declines with deviation from the optimum in males and females, respectively (i.e., $\sigma _\mathrm{m}$ and $\sigma _\mathrm{f}$ modulate the strength of selection in males and females).

### A sexually antagonistic locus on the PAR

As a baseline, we assume *z* is influenced by a locus (“trait locus” or “z-locus”) in the pseudo-autosomal region (PAR) of a sex chromosome that recombines with a male SDR with probability $r > 0$ per meiosis. We therefore initially consider an XY system, which under the random union of gametes model of mating is equivalent to a ZW system with the sex labels exchanged. This symmetry breaks down when male and female reproductive variances differ owing to the mating system; in that case, we model a ZW system explicitly (see the *Sexual antagonism strongly promotes ZW recombination evolution through W-linked suppressors* section).

Alleles at the trait locus have additive effects on phenotype, such that an individual’s trait value is given by $z = x_1 + x_2$, where $x_1$ is the allelic value carried on its maternally inherited X chromosome and $x_2$ is the allelic value carried on its paternally inherited chromosome (which is an X chromosome in females and a Y chromosome in males). Because alleles have the same phenotypic effect in both sexes but male and female fitness are maximized at different trait values ($-1$ in males and 1 in females), an allele that increases fitness in one sex can decrease fitness in the other; i.e., the trait locus is sexually antagonistic.

### Invasion of a cis-recombination suppressor

We investigate the fate of a new mutation arising within the PAR that fully suppresses recombination between the SDR and the *z*-locus (such as an inversion capturing both regions) when the trait locus is polymorphic for sexually antagonistic alleles. We consider two cases of sexually antagonistic polymorphism.

- We assume that two alleles, $\mathrm{A}$ and $\mathrm{a}$, segregate at the trait locus with effects $x_\mathrm{A}$ and $x_\mathrm{a}$, respectively, such that a balanced polymorphism is maintained at the trait locus (otherwise polymorphism would be transient and unlikely to be captured by a recombination suppressor). Without loss of generality, we assume $x_\mathrm{A} < x_\mathrm{a}$, so that the $\mathrm{A}$-allele tends to be male-beneficial and $\mathrm{a}$-allele female-beneficial.
- We focus on the situation where $x_\mathrm{A}$ and $x_\mathrm{a}$ have converged to a polymorphic evolutionary equilibrium via Kimura’s continuum-of-alleles model (Kimura, 1965); i.e., each mutation adds a small random deviation with mean zero to the allelic value it occurs on. In this model, sexually antagonistic selection can drive the gradual emergence of polymorphism via evolutionary branching (Online Appendix A.1; see Flintham et al., 2025 for more details about evolutionary branching on the PAR). This eventually results in the stable coexistence of two diverged allelic lineages whose equilibrium values we denote by $x_\mathrm{A}=x_\mathrm{A}^{*}$ and $x_\mathrm{a}=x_\mathrm{a}^{*}$ (Online Appendix A.2 explains how we characterized this polymorphism).

These two cases may reflect different timescales of evolution at the trait locus and the recombination modifier. Model (i) applies when mutations suppressing recombination and those influencing *z* arise on similar timescales. Model (ii) applies when mutations affecting allelic effects at the trait locus occur more frequently than mutations at recombination modifiers, so that the trait locus typically equilibrates before a new suppressor arises. Model (ii) may also capture a polygenic situation in which *z* is encoded by multiple PAR loci with fixed additive effects; we analyze such a model in Online Appendix B, where we obtain results analogous to those under the continuum-of-alleles model.

We initially assume the recombination suppressor appears on the Y chromosome (i.e., is Y-linked) and captures the SDR itself. Later in the *Sex chromosome evolution is more readily driven by X- than Y-linked suppressors under sexual antagonism* section, we consider a suppressor appearing on the X chromosome (i.e., X-linked) and capturing the homologous genomic region to the SDR on the X (equivalent modifiers in ZW systems are modeled in the *Sexual antagonism strongly promotes ZW recombination evolution through W-linked suppressors* section).

To investigate the evolution of recombination arrest, we first calculate the invasion fitness $W_r^\mathrm{Y}(x_\mathrm{a},x_\mathrm{A})$ of a rare Y-linked suppressor in a resident population where recombination otherwise occurs at rate *r* between the SDR and the trait locus, and where alleles with effect sizes $x_\mathrm{a}$ and $x_\mathrm{A}$ segregate at their equilibrium frequencies (in Online Appendix C.1, we characterize $W_r^\mathrm{Y}(x_\mathrm{a},x_\mathrm{A})$, which formally is defined as the long-term geometric mean of the average per-capita number of suppressor copies produced per demographic time step). When $W_r^\mathrm{Y}(x_\mathrm{a},x_\mathrm{A}) > 1$, the suppressor can invade the population; otherwise, it goes extinct with probability one (Harris, 1963).

## Results

### Sexually antagonistic polymorphism on the PAR generates male heterozygote advantage

**Figure 1:**
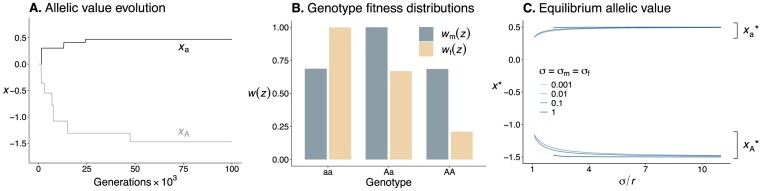
Evolution of allelic values at a sexually antagonistic locus on the PAR. Panel (A) shows the evolution of the allelic values $x_\mathrm{a}$ and $x_\mathrm{A}$ from individual-based simulations (Online Appendix A for details). Plot shows the allelic values encoded by the two most frequent alleles at a given generation in a simulation replicate (which correspond to alleles $\mathrm{a}$ and $\mathrm{A}$). These values diverge through the accumulation of recurrent mutations, until they converge at $x_\mathrm{a}=x_\mathrm{a}^{*}\approx 0.5$ and $x_\mathrm{A}=x_\mathrm{A}^{*}\approx -1.5$. Other parameters used in simulation: $N=10^4, \sigma =\sigma _\mathrm{m}=\sigma _\mathrm{f}=0.1, r = 0.005$, effects of new mutations are sampled from a normal distribution with mean 0 and standard deviation 0.25. Panel (B) shows the fitness ($w_\mathrm{m}(z)$ and $w_\mathrm{f}(z)$) of different genotypes ($\mathrm{a}\mathrm{a}$, $\mathrm{A}\mathrm{a}$ and $\mathrm{A}\mathrm{A}$) at generation $10^5$ of the simulation in panel (A), i.e., once allelic values have converged to their evolutionary stable values $(x_\mathrm{a}^{*},x_\mathrm{A}^{*})$. These show male heterozygote advantage. Panel (C) shows the evolutionary stable values $(x_\mathrm{a}^{*},x_\mathrm{A}^{*})$ (see Online Appendix A for calculation) as a function of the ratio between the strength of sexually antagonistic selection and the recombination rate ($\sigma /r$ where $\sigma =\sigma _\mathrm{m}=\sigma _\mathrm{f}$; recall $r < \sigma$ is the condition for disruptive selection in the PAR, hence why some curves do not cover the entire *x*-axis). Different curves show this for different strengths of $\sigma$. Regardless of the strength of sexually antagonistic selection, allelic values at the stable values are $x_\mathrm{a}^{*}\approx 0.5$ and $x_\mathrm{A}^{*}\approx -1.5$ unless $\sigma /r$ is close to 1 (which corresponds to where disruptive selection at the trait locus is weakest). Similar results are found when $\sigma _\mathrm{m}\ne \sigma _\mathrm{f}$ (see Supplementary Figure S1).

As a first step, we characterize the polymorphism that emerges through gradual evolution (continuum-of-alleles model; case (ii) in the *Invasion of a cis-recombination suppressor* section) at the trait locus whilst keeping recombination rate fixed at *r*. Using a numerical approach (Online Appendix A.2, in particular Online Appendix A.2.2), we find that for all parameters considered, the evolutionary equilibrium always consists of two alleles—$\mathrm{a}$ and $\mathrm{A}$—that respectively encode trait values $x_\mathrm{a}^{*}$ and $x_\mathrm{A}^{*}$ such that (i) in females, $\mathrm{a}\mathrm{a}$ homozygotes show the greatest fitness; (ii) in males, $\mathrm{A}\mathrm{a}$ heterozygotes show the greatest fitness (Figure 1A–B for an example, and see Figure 1C and Supplementary Figure S1 for $x_\mathrm{a}^{*}$ and $x_\mathrm{A}^{*}$ across a broad range of parameter sets). This occurs because one allele, $\mathrm{a}$ here, leads homozygotes to express a trait value close to the female optimum, while the other allele $\mathrm{A}$ leads homozygotes to express a trait value further from the male optimum than the one expressed by $\mathrm{A}\mathrm{a}$ heterozygotes (which occurs whenever $x_\mathrm{A} < -(2+x_\mathrm{a})/3$).

The “overshooting” of the male trait optimum by allele $\mathrm{A}$ occurs because once both alleles have begun diverging, $\mathrm{A}\mathrm{a}$ is the most frequent male genotype and hence selection on $\mathrm{A}$ favors these heterozygotes to be best adapted at the expense of $\mathrm{A}\mathrm{A}$ males. To see why, consider that once two alleles have diverged in their phenotypic effects, selection leads male-beneficial allele $\mathrm{A}$ to become overrepresented on the Y chromosome and hence in Y-bearing sperm, while female-beneficial allele $\mathrm{a}$ becomes overrepresented in X-bearing eggs. This increases the number of female (XX) offspring that are $\mathrm{a}\mathrm{a}$ homozygotes, and increases the number of male (XY) offspring that are $\mathrm{A}\mathrm{a}$ heterozygotes (meanwhile, the number of offspring that are $\mathrm{A}\mathrm{A}$ homozygotes decreases, owing to the low frequency of $\mathrm{A}$ in eggs). Consequently, selection favors an allelic value $x_\mathrm{A}$ for $\mathrm{A}$ that causes AA homozygotes to overshoot the male optimum because this $x_\mathrm{A}$ confers high male fitness in heterozygotes when combined with an allelic value $x_\mathrm{a}$ that confers high female fitness in homozygous form (e.g., with $\sigma _\mathrm{m}=\sigma _\mathrm{f}$, the combination of allelic values $x_\mathrm{A}=-1.5$ and $x_\mathrm{a}=0.5$ on average encodes the fittest phenotypes as they satisfy $x_\mathrm{A}+x_\mathrm{a}=-1$, the male optimum, and $x_\mathrm{a}+x_\mathrm{a}=1$, the female optimum). Overdominance here is restricted to males (Gavrilets & Rice, 2006, for a model assuming this on autosomes). This differs from the sex-averaged heterozygote advantage generated by dominance reversal for fitness (where homozygotes show the highest within-sex fitness but deleterious alleles are recessive in each sex; Connallon & Chenoweth, 2019; Fry, 2010), which is required to maintain balanced sexually antagonistic polymorphism on autosomes under weak selection (Kidwell et al., 1977).

While the evolution of male overdominance in the continuum-of-alleles model arises under the gradual evolution of allelic values through recurrent mutation, we also show in Online Appendix B that a similar pattern emerges when *z* is polygenic (encoded by multiple additive loci in the PAR) so long as recombination amongst loci is not too strong. In this case, selection quickly sorts standing variation to generate two predominant haplotypes: one linked to the X chromosome, with allelic effects summing to half the female optimum, and another linked to the Y chromosome, with effects overshooting the male optimum (in other words, X- and Y-linked haplotype values converge, respectively, to $x_\mathrm{a}^{*}$ and $x_\mathrm{A}^{*}$). Female homozygote advantage (favoring two copies of the X-linked haplotype) and male heterozygote advantage (favoring an X–Y haplotype combination) thus ensue.

### Sexual antagonism expedites sex chromosome evolution, but its potency is sensitive to the mating system

Next, we characterize the fate of a recombination modifier on the Y chromosome that arises as a single copy and fully suppresses recombination between the SDR and the trait locus; which is polymorphic for alleles $\mathrm{A}$ and $\mathrm{a}$. In Online Appendices C.1–C.2, we show that across combinations of allelic effects satisfying $x_\mathrm{a} > x_\mathrm{A}$, the invasion fitness of such a suppressor always exceeds unity ($W_r^\mathrm{Y}(x_\mathrm{a},x_\mathrm{A}) > 1$) if it captures allele $\mathrm{A}$ and is less than unity ($W_r^\mathrm{Y}(x_\mathrm{a},x_\mathrm{A}) < 1$) if it captures allele $\mathrm{a}$. That is, selection favors recombination suppressors capturing $\mathrm{A}$ and disfavors suppressors capturing $\mathrm{a}$ (as previously shown for small-effect recombination modifiers; see the condition for selection to favor recombination suppression in Otto, 2014 with $R_m=0$, and consistent with results from full suppressor models that constrain allelic values such that $x_\mathrm{A}$ is strictly male-beneficial, e.g., Charlesworth & Charlesworth, 1980; Nei, 1969; Olito & Abbott, 2025). In addition, we find that selection for recombination arrest is greatest ($W_r^\mathrm{Y}(x_\mathrm{a},x_\mathrm{A})$ largest) when the resident recombination rate (*r*) is largest (see also Olito & Abbott, 2025) and is especially strong when alleles have evolved to their evolutionary equilibrium (i.e., when $x_\mathrm{a}= x_\mathrm{a}^{*}$ and $x_\mathrm{A}=x_\mathrm{A}^{*}$, see Online Appendix C.2 for discussion).

The tempo of recombination evolution, however, depends not just on whether selection can favor a rare suppressor, but also on the likelihood that such a mutant evades stochastic loss and goes on to fix in the population. To quantify this, we first calculate the (unconditional) probability of invasion $\Psi ^{\mathrm{Y}}$ of a Y-linked suppressor: the probability that a suppressor arises in a chromosome carrying allele $\mathrm{A}$ and that this suppressor does not go extinct in the long run, i.e., $\Psi ^{\mathrm{Y}}= \hat{p}_{ \mathrm{m}}^{\mathrm{Y}} \Psi ^{\mathrm{Y}}_\mathrm{A}$, where $\Psi ^{\mathrm{Y}}_\mathrm{A}$ is the invasion probability of the suppressor conditional on it capturing $\mathrm{A}$ and $\hat{p}_{ \mathrm{m}}^{\mathrm{Y}}$ is the equilibrium frequency of $\mathrm{A}$ in sperm (see Online Appendix C.3.1 for details, and also Olito & Abbott, 2025 for a similar approach). Across all parameters tested, suppressors that do not go extinct (i.e., successfully invade) always go on to fix in the population; in other words, suppressors do not form balanced polymorphisms (Online Appendix C.3.2 for details). The probability of invasion, $\Psi ^{\mathrm{Y}}$, is therefore equivalent to the probability of fixation, and this allows us to leverage $\Psi ^{\mathrm{Y}}$ to compute a suppressor’s expected time to substitution, $\tau$ (the expected waiting time to arise and then fix in a population, Online Appendix C.3.2 for details). We use $\tau$ as a measure of the rate of recombination evolution.

We first perform this analysis under the life cycle described in the *Lifecycle and fitness* section, where mating is modeled as a random-union-of-gametes model (such that the offspring distribution of a rare recombination suppressor is Poisson with mean given by equation C-4). We calculated $\Psi ^{\mathrm{Y}}$ for different allelic values $x_\mathrm{a}$ and $x_\mathrm{A}$, and parameters $\sigma _\mathrm{m}$, $\sigma _\mathrm{f}$ and *r* (Online Appendix C.3.1 for details). As expected from the results for the magnitude of $W_r^\mathrm{Y}(x_\mathrm{a},x_\mathrm{A})$ (Online Appendix C.2), invasion probability $\Psi ^{\mathrm{Y}}$ is especially large when allelic values are at their evolutionary equilibrium (i.e., when $x_\mathrm{a}=x_\mathrm{a}^{*},x_\mathrm{A}=x_\mathrm{A}^{*}$, Supplementary Figure S4 and Figure 3A compare dotted and solid lines), and greatest when sexual antagonism is strong and the resident recombination rate is high ($\sigma =\sigma _\mathrm{m}=\sigma _\mathrm{f}$ and *r* large, Figure 3A).

More generally, unless allelic values are very weakly diverged ($x_\mathrm{a}$ and $x_\mathrm{A}$ similar) or sexually antagonistic selection is very weak ($\sigma$ very small), $\Psi ^{\mathrm{Y}}$ is high compared to the neutral expectation in a large population (as also suggested by Olito & Abbott, 2025, by comparing the scale of *Y*-axes in their Figure 2A–C). For example, with allelic values $x_\mathrm{a}=0.5$ and $x_\mathrm{A}=-0.5$ and loose linkage $r=0.5$, $\Psi ^{\mathrm{Y}}\approx 0.1$ for $\sigma =0.1$ and $\Psi ^{\mathrm{Y}}\approx 0.01$ for $\sigma =0.01$ (Figure 3A). A neutral suppressor would fix with a similar probability only in very small populations (with 10 and 100 males or less for $\sigma =0.1$ and $\sigma =0.01$, respectively). In line with this, the expected time to substitution for a recombination suppressor is orders of magnitude shorter if it captures a sexually antagonistic locus than for a neutral suppressor (e.g., with reasonable suppressor mutation rates and $r=0.5$, a substitution can be expected to occur roughly 1,000 times faster than at neutrality when $\sigma =0.1$, and around 100 times faster when $\sigma =0.01$, Figure 3B).

**Figure 2:**
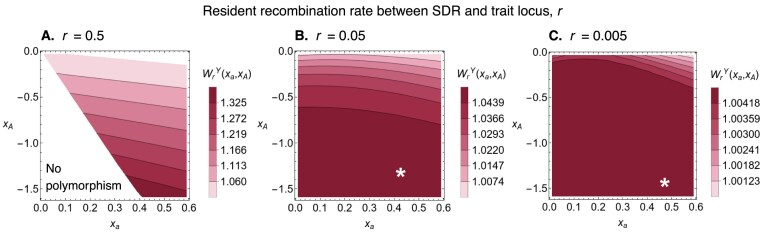
Invasion fitness of a Y-linked suppressor capturing a trait locus with alleles encoding $x_\mathrm{a}$ and $x_\mathrm{A}$. Plots show the invasion fitness, $W_r^\mathrm{Y}(x_\mathrm{a},x_\mathrm{A})$ (equation C-4 in Online Appendix C.2, see also Appendix C.1 for calculation details), of a Y-linked recombination suppressor capturing allele $\mathrm{A}$ at the trait locus as a function of the allelic values $x_\mathrm{a}$ and $x_\mathrm{A}$. Different panels show results for different levels of resident recombination between the trait locus and the SDR, *r*. White stars show the location of the evolutionary stable allelic values that would emerge through gradual evolution $(x_\mathrm{a}^{*},x_\mathrm{A}^{*})$ (there is no star in panel (A), because sexual antagonism does not produce disruptive selection here: $r > \sigma$, with balancing selection thus requiring large-effect mutations; see equation A-2 in Online Appendix A.1). Non-colored space indicates that a suppressor cannot invade (which is because these conditions do not lead to a balanced polymorphism for alleles $\mathrm{A}$ and $\mathrm{a}$ at the trait locus). Other parameters used $\sigma =\sigma _\mathrm{m}=\sigma _\mathrm{f}=0.1$ (see Supplementary Figure S3 for cases where $\sigma _\mathrm{m}\ne \sigma _\mathrm{f}$, and Supplementary Figure S2 for the selection and dominance coefficients of alleles A and a in the notation of Kidwell et al. 1977).

**Figure 3:**
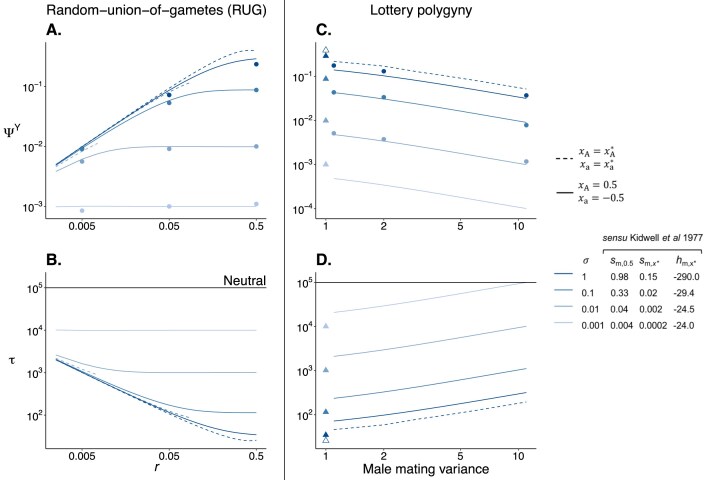
Invasion probability and waiting times of Y-linked suppressors. Panel (A) shows the unconditional invasion probability of a newly arisen Y-linked suppressor, $\Psi ^{\mathrm{Y}}$, under the random-union-of-gametes (RUG) model (Online Appendix C.3.1 for calculation) according to the resident recombination rate (*r*). Dashed lines correspond to invasion probabilities when allelic values have converged to their evolutionary stable values ($x_\mathrm{a}^{*}, x_\mathrm{A}^{*}$) and are therefore shown only for the range of parameters where evolutionary branching occurs. Solid lines correspond to invasion probabilities when allelic values have diverged, but not so much that males show heterozygote advantage: $x_\mathrm{a}=0.5$ and $x_\mathrm{A}=-0.5$. Dots show results from individual-based simulations with $x_\mathrm{a}=0.5$ and $x_\mathrm{A}=-0.5$ (which correspond to proportion of replicate simulations in which a Y-suppressor fixed, Online Appendix C.3.2). Different colors correspond to different strengths of selection at the trait locus ($\sigma =\sigma _\mathrm{m}=\sigma _\mathrm{f}$). Other measures of the strength of selection at the trait locus (following the convention of Kidwell et al., 1977) are also given in the figure table: (1) the male homozygous selection coefficient, $s_\mathrm{m}=[w_\mathrm{m}(2x_\mathrm{A})-w_\mathrm{m}(2x_\mathrm{a})]/w_\mathrm{m}(2x_\mathrm{A})$, for allele $\mathrm{a}$ (where $s_{\mathrm{m},0.5}$ refers to the case $x_\mathrm{a}=0.5$, $x_\mathrm{A}=-0.5$, and $s_{\mathrm{m},x^{*}}$ to the case $x_\mathrm{a}=x_\mathrm{a}^{*}$, $x_\mathrm{A}=x_\mathrm{A}^{*}$); (2) the male dominance coefficient for allele $\mathrm{a}$ when $x_\mathrm{a}=x_\mathrm{a}^{*}$ and $x_\mathrm{A}=x_\mathrm{A}^{*}$, $h_{\mathrm{m},x^{*}}=[w_\mathrm{m}(2x_\mathrm{A})-w_\mathrm{m}(x_\mathrm{a}+x_\mathrm{A})]/[w_\mathrm{m}(2x_\mathrm{A})-w_\mathrm{m}(2x_\mathrm{a})]$ – negative values for $h_\mathrm{m}$ indicate male fitness overdominance, and we see large absolute values here because homozygotes show similar fitness when alleles are at their evolutionary stable values; i.e., $s_{\mathrm{m},x^{*}}$ is very small. Panel (B) shows the expected waiting time $\tau$ for a Y-linked suppressor to substitute into a population of $10^4$ males (i.e., $N=2\times 10^4$) with mutation rate $\mu =10^{-5}$ (these are equivalent parameters to those considered by Lenormand & Roze, 2022, their Figure 2, see Online Appendix C.3.2 for calculation and further discussion of mutation rates). The same parameter values are used as in panel (A). Horizontal black line shows the expected waiting time for a purely neutral Y-linked allele ($1/\mu$). Panels (C) and (D) show the same output as (A) and (B) but for a model of lottery polygyny. In these panels, we assume loose linkage $r=0.5$ between the SDR and trait locus, and vary the degree of variance in male mating success (Online Appendix C.3.3 for calculation; note that evolutionary branching only occurs when $\sigma > 0.5$ here). Triangles show equivalent $\Psi ^{\mathrm{Y}}$ and $\tau$ values under the RUG model (empty and filled triangles for $x_\mathrm{a}=x_\mathrm{a}^{*}, x_\mathrm{A}=x_\mathrm{A}^{*}$ and $x_\mathrm{a}=0.5$, $x_\mathrm{A}=-0.5$ cases, respectively). Note the discrepancy between these values and those for polygyny with low male mating variance are due to the increase in drift produced by monoandry (females mating singly) in the lottery polygyny model.

The random-union-of-gametes model is a good approximation for large outbred populations where individuals of both sexes mate many times, but not for the many species experiencing sex-specific reproductive variance or skew (Andersson, 1994; Clutton-Brock & Parker, 1992; Shuker & Simmons, 2014). To capture such a situation, we now consider a lifecycle that begets a lottery polygyny mating system (Lee et al., 2008; Nunney, 1993). Briefly, we assume females mate singly but males can mate multiply (full details of this life cycle are given in Online Appendix C.3.3). A male’s likelihood of mating with each female is determined by his relative mating competitiveness, which is a random value assigned to him upon reaching adulthood (e.g., capturing his biological “condition,” Wilson & Nussey, 2010, which we assume is independent of genotype here, to facilitate comparison between models). Male reproductive variance therefore emerges because on average some males mate with more females, and this effect can be tuned by modifying the distribution of male mating competitiveness, with variance in mating competitiveness modulating the distribution of male mating and reproductive success (Lee et al., 2008). We detail how we calculate the probability of invasion $\Psi ^{\mathrm{Y}}$ of a Y-linked suppressor under this model in Online Appendix C.3.3.

Comparison between $\Psi ^{\mathrm{Y}}$ calculated under this model and under the random-union-of-gametes reveals that Y-linked recombination suppressors are less likely to invade under lottery polygyny (Figure 3C). This is because high variability in male mating success elevates the probability that a male by chance mates no females and so produces no offspring. This in turn exacerbates the probability that the lineage carrying a recombination suppressor suffers stochastic extinction. Consequently, adaptive (i.e., those capturing allele $\mathrm{A}$) suppressors will fix with probabilities similar to neutral suppressors in much larger populations under lottery polygyny than under the random-union-of-gametes model—especially when males show large variability in their mating success. For example, where male mating variance is around 10 (which is well within empirical estimates; Wade & Shuster, 2004 Table 1), population sizes of $10^3$ males will yield similar neutral and adaptive fixation probabilities when $r=0.5$ and $\sigma =0.01$. Accordingly, adaptive recombination suppression evolves more slowly in the presence of high male reproductive variance (Figure 3D), and even on the same timescale as neutral suppression if sexual antagonism is sufficiently weak (Figure 3D, $\sigma =0.001$).

### Sex chromosome evolution is more readily driven by X- than Y-linked suppressors under sexual antagonism

We next investigate the potential for recombination evolution to occur through X- vs. Y-linked modifiers. Divergence between sex chromosomes may just as well be mediated by recombination modifiers fixing on the X as the Y (e.g., Charlesworth & Charlesworth, 2005; Lenormand & Roze, 2022; Olito & Abbott, 2025, see also Lenormand, 2003; Otto, 2014 for gradual suppression models that are agnostic as to the genomic location of modifiers), but some authors have suggested that X-driven suppression is unlikely because X-linked suppressors will be more weakly selected than those on the Y (see below, and Blackmon & Brandvain, 2017; Charlesworth & Charlesworth, 1980; Olito & Abbott, 2025). To study recombination evolution on the X chromosome, we now consider the invasion of a suppressor on the X chromosome across different allelic values ($x_\mathrm{a}$ and $x_\mathrm{A}$, including their evolutionary equilibrium $x_\mathrm{a}^{*}$ and $x_\mathrm{A}^{*}$) and mating systems (random-union-of-gametes and polygyny models). We assume this modifier fully suppresses recombination in both males and heterozygous females (as for a chromosomal inversion, Kirkpatrick, 2010).

We first calculate the invasion fitness of an X-linked suppressor, $W_{r}^\mathrm{X}(x_\mathrm{a},x_\mathrm{A})$ (in Online Appendix D.1). Analysis of $W_{r}^\mathrm{X}(x_\mathrm{a},x_\mathrm{A})$ shows that such a suppressor is favored (i.e., $W_{r}^\mathrm{X}(x_\mathrm{a},x_\mathrm{A})> 1$) for any parameters $\sigma _\mathrm{m}$, $\sigma _\mathrm{f}$, and *r*, and allelic values $x_\mathrm{a}$ and $x_\mathrm{A}$ such that $\mathrm{a}$ and $\mathrm{A}$ are maintained under balancing selection, if and only if the suppressor captures female-beneficial allele $\mathrm{a}$. This is because linking allele $\mathrm{a}$ to the SDR homologue on the X chromosome ensures that males always transmit this allele to their female offspring, who then enjoy higher fitness as a result. As with Y-linked suppression, selection favors an X-linked suppressor most strongly (i.e., $W_{r}^\mathrm{X}(x_\mathrm{a},x_\mathrm{A})$ is largest) when recombination in males is frequent (*r* large), with selection especially strong when alleles are at their equilibrium values ($x_\mathrm{a}^{*}$ and $x_\mathrm{A}^{*}$) (Supplementary Figure S5).

Selection therefore readily favors X-linked suppression, but we find it does so less strongly than for suppression on the Y (i.e., $W_{r}^\mathrm{X}(x_\mathrm{a},x_\mathrm{A}) < W_r^\mathrm{Y}(x_\mathrm{a},x_\mathrm{A})$, Supplementary Figure S6A, consistent with the results of Blackmon & Brandvain, 2017; Charlesworth & Charlesworth, 1980). The selective advantage of an X-linked suppressor is approximately three times weaker than the advantage of a Y-linked suppressor when selection for recombination suppression is weak ($W_{r}^\mathrm{X}(x_\mathrm{a},x_\mathrm{A})-1\approx [W_r^\mathrm{Y}(x_\mathrm{a},x_\mathrm{A})-1]/3$ when $\sigma _\mathrm{f}$ and $\sigma _\mathrm{m}$ small or *r* small). This is primarily because the X spends two-thirds of its time in females where recombination suppression is inconsequential to offspring fitness (as chromosome segregation in females is random with respect to offspring sex, Blackmon & Brandvain, 2017; Charlesworth & Charlesworth, 1980). This dilutes the fitness advantage of X-linked recombination suppressors relative to those on the Y, which are passed strictly through males. An additional penalty to X-linked suppression arises for non-equilibrium allelic values where males do not show heterozygote advantage and thus ubiquitously favor allele $\mathrm{A}$, as here an X-suppressor capturing allele $\mathrm{a}$ will confer decreased fitness when carried in males.

Next, we calculate the probability of invasion $\Psi ^{\mathrm{X}}$ for an X-linked suppressor of recombination and compare it with its Y-linked counterpart $\Psi ^{\mathrm{Y}}$ (Online Appendices D.2.1–D.3 for full description of invasion probabilities for X-linked suppressors; note that some of the following results contradict those in Appendix C of Olito & Abbott, 2025, which is due to a technical error in Olito & Abbott, 2025 that is corrected here, see Online Appendix D.2.2 for details). Where the offspring distribution of a rare X-linked suppressor is Poisson (as expected under the random-union-of-gametes model), we find that such a suppressor is less likely to invade than a suppressor arising on the Y, i.e., $\Psi ^{\mathrm{X}} < \Psi ^{\mathrm{Y}}$ (Supplementary Figure S6B). This is unsurprising given that selection favoring X-linked suppression is weaker (i.e., $W_{r}^\mathrm{X}(x_\mathrm{a},x_\mathrm{A}) < W_r^\mathrm{Y}(x_\mathrm{a},x_\mathrm{A})$).

**Figure 4:**
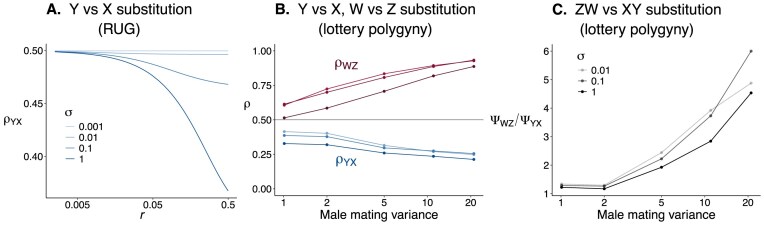
Likelihood of heterogametic- vs. homogametic-linked suppression and pace of evolution in XY vs. ZW systems. Plots show the probability that a substitution by a recombination suppressor is on the heterogametic vs. homogametic chromosome (Y- vs. X-linked: $\rho _\mathrm{YX}=\Psi ^{\mathrm{Y}}/(\Psi ^{\mathrm{Y}}+3\Psi ^{\mathrm{X}}_{ })$ or W- vs. Z-linked: $\rho _\mathrm{WZ}=\Psi ^{\mathrm{W}}/(\Psi ^{\mathrm{W}}+3\Psi ^{\mathrm{Z}}{}{}$), Online Appendices C.3.1 and D.2.1 for calculation of $\Psi ^{\mathrm{X}}_{ }$ and $\Psi ^{\mathrm{Y}}$ in the random-union-gametes model, respectively). Panel (A) shows $\rho _\mathrm{YX}$ under the random-union-of-gametes (RUG) model for different strengths of sexually antagonistic selection ($\sigma =\sigma _\mathrm{m}=\sigma _\mathrm{f}$, see also Figure 3 for other measures of the strength of selection) and resident recombination rate *r*. Lines show results for allelic values $x_\mathrm{a}=0.5, x_\mathrm{A}=-0.5$. Note that in this model, XY and ZW systems are interchangeable ($\rho _\mathrm{YX}=\rho _\mathrm{WZ}$). Panel (B) shows the corresponding $\rho _\mathrm{YX}$ (blue curves and dots) and $\rho _\mathrm{WZ}$ (red curves and dots) values but for the polygyny model with loose linkage ($r=0.5$) and different strengths of male mating variance. Here, fixation probabilities are estimated from individual-based simulations (colored dots are individual estimates). Horizontal line marks $\rho _\mathrm{YX}=0.5$. Panel (C) shows ratio between the overall substitution rate in ZW systems relative to XY systems, $\Psi ^{\mathrm{Z}\mathrm{W}}/\Psi ^{\mathrm{X}\mathrm{Y}}$ where $\Psi ^{\mathrm{Z}\mathrm{W}}=\Psi ^{\mathrm{W}}+3\Psi ^{\mathrm{Z}}{}{}$ and $\Psi ^{\mathrm{X}\mathrm{Y}}=\Psi ^{\mathrm{Y}}+3\Psi ^{\mathrm{X}}_{ }$, for different male mating variances. Dots are again estimated from individual-based simulations. See Figure 3 for corresponding parameter values for selection at sexually antagonistic loci in Kidwell et al. (1977) notation.

Nevertheless, the discrepancy between X and Y invasion probabilities remains moderate—equal to or less than threefold (Supplementary Figure S6B)—and can therefore be more than compensated for by stronger mutational input of X-linked inversions: When recombination suppressors arise with equal per-base rates in males and females, new suppressors are three times more likely to arise on the X than Y owing to the X’s larger population size. Therefore, the probability that a recombination suppression event (i.e., substitution by a suppressor) is due to a Y- rather than an X-linked modifier is $\rho _\mathrm{YX}=\Psi ^{\mathrm{Y}}/(\Psi ^{\mathrm{Y}}+3\Psi ^{\mathrm{X}})$ (provided suppressors arise rarely). Plugging in our values for $\Psi ^{\mathrm{Y}}$ and $\Psi ^{\mathrm{X}}$ into $\rho _\mathrm{YX}$ reveals that recombination suppression is in fact equally likely to be X- or Y-linked when sexual antagonism is weak or initial linkage between the SDR and trait locus is tight (i.e., $\rho _\mathrm{YX}=0.5$ when *r* or $\sigma$ small, Figure 4A), because the threefold higher selective advantage to Y-linked suppressors cancels with the discrepancy in mutational input. Otherwise, we find X-linked suppression is more probable (e.g., $\rho _\mathrm{YX}< 0.5$ with $r=0.5$ and $\sigma \ge 0.1$ for $x_\mathrm{a}=0.5, x_\mathrm{A}=-0.5$ and $x_\mathrm{a}=x_\mathrm{a}^{*}, x_\mathrm{A}=x_\mathrm{A}^{*}$, Figure 4A). This is because when sexual antagonism and prior recombination are strong ($\sigma$ and *r* large), the selective advantage to Y-linked suppression is constrained by the combined effects of (i) beneficial alleles tending to be more recessive under Gaussian fecundity, and (ii) the excess of suppressor-carrying males who are $\mathrm{A}\mathrm{a}$ heterozygotes (relative to X-linked suppressor-carrying female $\mathrm{a}\mathrm{a}$ homozygotes, see the *Sexually antagonistic polymorphism on the PAR generates male heterozygote advantage* section), who therefore experience a weaker benefit of guaranteed paternal inheritance of allele $\mathrm{A}$. Y-linked suppressors thus show a below-threefold selective advantage relative to X-linked suppressors and so a lower substitution rate ($\rho _\mathrm{YX}< 0.5$).

Computing $\rho _\mathrm{YX}$ under lottery polygyny shows that recombination suppression is even more likely to be a consequence of X-linked suppressors under this mating system (Figure 4B). In particular, when male reproductive variance is high, recombination arrest among sex chromosomes is expected to be mostly due to X-linked factors (e.g., with a variance in the number of mates among males of around 10, $\rho \approx 0.25$, Figure 4B). This is because male reproductive variance has less influence on the level of drift experienced by X-linked than by Y-linked mutations so that, all else equal, X-linked suppressors are less susceptible to stochastic extinction than Y-linked suppressors here. By contrast, neutral substitution rates are always equal on the X and Y (as differences in mutational pressure and fixation probabilities balance out), leading to equivalent X–Y contributions to sex chromosome evolution through the fixation of neutral suppressors.

### Sexual antagonism strongly promotes ZW recombination evolution through W-linked suppressors

In populations showing equivalent reproductive variance in males and females, our XY random-union-of-gametes model also exactly captures ZW sex-determination. Our results therefore predict equal, or mildly Z-biased, contributions to sex chromosome evolution in such populations ($\rho _\mathrm{WZ}=\Psi ^{\mathrm{W}}/(\Psi ^{\mathrm{W}}+3\Psi ^{\mathrm{Z}})\approx 0.5$ or $\rho _\mathrm{WZ}< 0.5$). However, in species with sex-specific reproductive variance, evolutionary rates on different heterogametic (X vs. Z) and homogametic (Y vs. W) sex chromosomes can diverge. To investigate the consequences of this for sex chromosome evolution, we ran simulations of our polygyny model with a ZW system, i.e., where females carry a dominant SDR and males are homozygous for the SDR homologue (Online Appendix D.3).

In ZW systems, elevated male reproductive variance leads to a very strong W-bias in the contribution to recombination arrest (Figure 4B), owing to strong genetic drift through males dampening the efficacy of selection favoring Z-linked modifiers. Fixing suppressors are expected to almost always be W-linked when reproductive variance is high (e.g., with a variance in the number of mates among males of around 10, $\rho _\mathrm{WZ}\approx 0.9$, Figure 4B). The degree of W-bias is greater than the degree of X-bias for the same mating system because (i) suppressors on the W chromosome are under stronger selection than those on the X, and (ii) unlike X chromosomes, W chromosomes are never present in males, and so W-linked suppressors do not experience any inflation in drift due to polygyny.

Comparing the time to substitution for a recombination suppressor in a ZW system (which is inversely proportional to $\Psi ^{\mathrm{Z}\mathrm{W}}=3\Psi ^{\mathrm{Z}}+\Psi ^{\mathrm{W}}$) to that in an XY system (inversely proportional to $\Psi ^{\mathrm{X}\mathrm{Y}}=3\Psi ^{\mathrm{X}}+ \Psi ^{\mathrm{Y}}$), we see that in the presence of excess male reproductive variance, sexual antagonism drives faster recombination suppression in ZW systems ($\Psi ^{\mathrm{Z}\mathrm{W}}$ is greater than $\Psi ^{\mathrm{X}\mathrm{Y}}$, Figure 4C). This is again because the Y chromosome is more strongly selected than the Z-chromosome (i.e., has larger fixation probability under random-union-of-gametes model; $\Psi ^{\mathrm{Y}} > \Psi ^{\mathrm{X}}=\Psi ^{\mathrm{Z}}$), such that the effect of polygyny in eroding the efficacy of selection for modifiers on the Y disproportionately diminishes total substitution rates in XY systems relative to ZW.

### Deleterious variation can hinder fixation on X and Z chromosomes

As a final step, we consider the joint effects of sexual antagonism and deleterious variation in the PAR on selection for recombination suppression. To do this, we ran multilocus simulations of a sex chromosome region comprising the SDR (or its homologue) and a section of the PAR containing a (i) single sexually antagonistic locus (with alleles $\mathrm{A}$ and $\mathrm{a}$ segregating) and (ii) a fixed number, *L*, of loci where deleterious alleles can appear (see Online Appendix E for full simulation details). Alleles at the sexually antagonistic and deleterious loci contribute multiplicatively to fitness, with each deleterious allele encoding a proportional reduction to fecundity of *s* and $hs$ in homozygous and heterozygous form, respectively. Simulations are run until mutation-selection-drift balance at deleterious loci is reached before a recombination modifier is introduced. Allele $\mathrm{A}$, meanwhile, is assumed to be at its equilibrium frequency. We then consider the fate of a suppressor that captures the entire chromosomal region with either the SDR (constituting a Y- or W-linked suppressor) or the SDR homologue (constituting an X- or Z-linked suppressor). As before, this suppressor inhibits recombination when in heterozygous form, as for an inversion. We estimate fixation probabilities for each type of suppressor (X, Y, Z, W) across a range of parameters characterizing deleterious ($s, h$) and sexually antagonistic ($\sigma$) variation.

**Figure 5:**
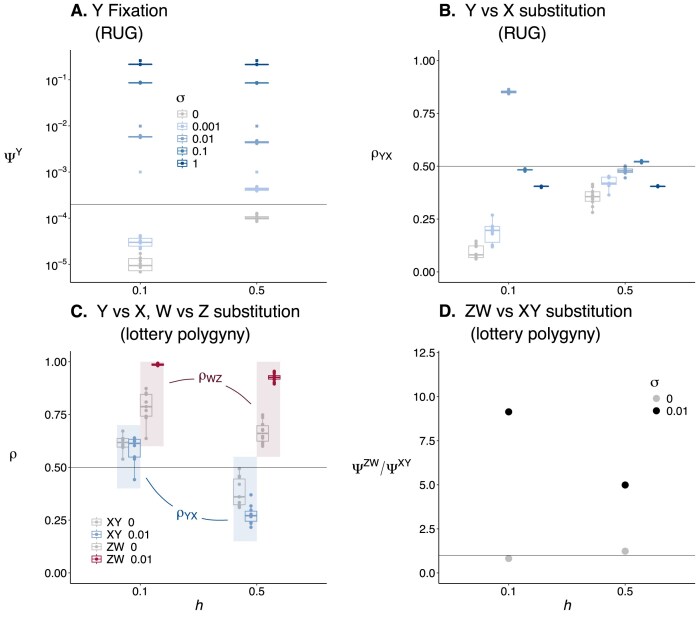
Selection for recombination suppression when deleterious and sexually antagonistic alleles co-segregate. Panel (A) shows the fixation probabilities estimated from individual-based simulations of a Y-linked suppressor capturing both a sexually antagonistic locus and $L=100$ deleterious loci at mutation-selection balance (Online Appendix E for simulation details). Results are shown for selection coefficient $s=0.025$ and dominance coefficients of either $h=0.1$ and $h=0.5$ at all deleterious loci (see *x*-axis). Each dot is an estimate for a single replicate population in a simulation of the random union-of-gametes model. Boxplot and dot colors correspond to different strengths of sexually antagonistic selection ($\sigma$). Squares show the invasion probability in the absence of deleterious variation (Figure 3A). Horizontal line is the neutral fixation probability for a Y-linked mutation ($1/(N/2)$), where $N=10^4$. Recombination is scaled such that $r=0.3$ between the sexually antagonistic locus and SDR (Online Appendix E). Panel (B) shows the probability that a substitution by a recombination suppressor is Y- vs. X-linked, $\rho _\mathrm{YX}$, from the same simulation setup as in panel A (here XY and ZW systems are interchangeable: $\rho _\mathrm{YX}=\rho _\mathrm{WZ}$). Horizontal line marks $\rho _\mathrm{YX}=0.5$. Panel (C) shows the probability a substitution is on the heterogametic chromosome ($\rho _\mathrm{YX}$ or $\rho _\mathrm{WZ}$) in the lottery polygyny model with high male mating variance (variance $\approx 20$) for different strengths of sexually antagonistic selection and dominance coefficients at the deleterious loci. Boxes and points in blue shaded regions show results for an XY system (showing $\rho _\mathrm{YX}$) and boxes and points in red shaded regions show results for a ZW system (showing $\rho _\mathrm{WZ}$), gray and colored boxes/points correspond to $\sigma =0$ and $\sigma =0.01$ cases, respectively. Panel (D) shows overall substitution rates in XY and ZW systems with same parameters as in C, where each line is the average of all populations in the ZW simulations relative to the average of all populations in XY simulations (horizontal line marks $\Psi ^{\mathrm{Z}\mathrm{W}}/\Psi ^{\mathrm{X}\mathrm{Y}}=1$). See Figure 3 for corresponding parameter values for selection at sexually antagonistic loci in Kidwell et al. (1977) notation.

In line with previous work (Charlesworth & Olito, 2024; Olito & Abbott, 2025; Olito et al., 2024; Roze & Lenormand, 2025), we find that in the absence of sexual antagonistic selection, deleterious variation generally generates below-neutral fixation probabilities for recombination modifiers on the Y or W chromosomes (Figure 5A and Supplementary Figure S7A gray dots; above-neutral probabilities require deleterious alleles have weak effects and are highly recessive—$sh$ is very small—leading to the “sheltering effect,” Jay et al., 2024; see Roze & Lenormand, 2025 for in-depth treatment). Furthermore, we also observe a pattern of below-neutral fixation of X- and Z-linked modifiers, although this reduction is less severe than for modifiers on the Y or W (Supplementary Figure S7B gray dots). This discrepancy is due to Muller’s ratchet acting on the backgrounds captured by “lucky” Y- and W-modifiers, which accelerates the accumulation of deleterious variants and hampers suppressor fixation (Roze & Lenormand, 2025). Muller’s ratchet is not seen in backgrounds captured by X- and Z-modifiers, as they still experience recombination in homozygotes.

When deleterious and sexually antagonistic alleles co-segregate, we find lower fixation probabilities relative to when sexually antagonistic alleles segregate in isolation (Figure 5A). Deleterious variation hinders the fixation of suppressors here because the fitness benefit of picking up a beneficial sexually antagonistic allele (allele $\mathrm{a}$ for X- and Z-linked suppressors, and allele $\mathrm{A}$ for Y- and W-linked suppressors) can be masked if the suppressor is “unlucky,” i.e., it also captures a particularly loaded genetic background, or accumulates deleterious alleles during its segregation. Nevertheless, fixation probabilities for suppressors capturing a sexually antagonistic locus remain well above those for suppressors capturing deleterious loci alone, even when sexually antagonistic selection is weak. In other words, suppressors predominantly experience selection according to the allele they capture at the sexually antagonistic locus, which is because these loci account for a greater proportion of the variance in fitness among genetic backgrounds than deleterious loci at mutation-selection-drift balance. Waiting times for the substitution of recombination suppressors will thus tend to be orders of magnitude shorter in the presence of sexual antagonism.

We also find that unbiased, X/Z-biased, and (more rarely) W/Y-biased substitution rates are all possible when deleterious and sexually antagonistic variation co-exists (Figure 5B and Supplementary Figure S7C). In general, when male and female reproductive variances are equal, substitution rates are similar for modifiers arising on the hetero- and homogametic sex chromosomes if sexual antagonism is appreciable ($\rho \approx 0.5$, in line with results presented in the *Sex chromosome evolution is more readily driven by X- than Y-linked suppressors under sexual antagonism* section). Meanwhile, X/Z-biased substitution is observed when sexual antagonism is very weak or absent, in which case fixation is driven by the capture of deleterious variation. Here, a combination of opposing effects: on one hand, Muller’s ratchet on the Y/W, and on the other, higher starting frequencies for Y/W- than X/Z-linked suppressors, lead to relatively similar fixation probabilities between hetero- and homogametic suppressors, and so overall to a X/Z-biased substitution rate (the exception being where $sh$ is low enough to drive strongly Y/W-biased fixation rates through the sheltering effect, Supplementary Figure S7C with $s=0.005, h=0.1$).

Finally, our previous findings of divergence in genomic patterns between XY and ZW systems under male-biased reproductive variance also hold in the presence of deleterious variation. First, sexual antagonism can again lead to homogametic-biased (X-biased) substitution in XY systems, but heterogametic-biased (W-biased) substitution in ZW systems (Figure 5C). Second, overall substitution rates in ZW systems are also larger than those in XY systems (Figure 5D). These discrepancies between XY and ZW systems were either weak or absent when suppression evolved without sexual antagonism (Figure 5C–D).

## Discussion

### Different timescales for sex chromosome evolution with vs. without sexual antagonism

Our analyses indicate that recombination suppressors capturing sexually antagonistic loci generally have a substantially higher probability of invasion (and correspondingly lower expected time to substitution) compared with neutral suppressors or lucky suppressors capturing few deleterious alleles. Significant between-taxa variation exists in the rate at which recombination suppression evolves along sex chromosomes (Ma & Rovatsos, 2022). Our results thus suggest that systems evolving at different rates are being driven by different forces: fast suppression evolution associated with the segregation of sexually antagonistic alleles, while slow evolution proceeds due to the rare fixation of neutral or lucky suppressors. Another finding of our study is that the capture of a sexually antagonistic locus can appreciably influence the fixation probability of a recombination suppressor even when sex-specific selection is very weak and thus difficult to detect empirically. Because such loci may persist readily as balanced polymorphisms when they are located in the PAR, sexual antagonism can thus play a role in driving sex chromosome evolution even when its other effects are imperceptible.

Sexually antagonistic selection is particularly potent in driving recombination suppression when males show heterozygote advantage (for XY systems), which we have shown to be the evolutionary equilibrium for an antagonistic locus on the PAR. Alleles may reach this equilibrium through repeated small-effect mutations, as in the continuum-of-alleles model, though this process may be slow. But male heterozygote advantage could evolve much more rapidly, for instance, through large-effect mutations—in fact, mutations that overshoot the male optimum in a single step have the highest invasion fitness and are thus the most likely to invade—or through the evolution of standing variation in a polygenic trait encoded on the PAR (Online Appendix B). Male heterozygote advantage is therefore a plausible outcome of sexually antagonistic selection on the PAR, which in turn facilitates the evolution of recombination suppression.

### Recombination arrest may be driven by suppressors on heterogametic or homogametic sex chromosomes

Our analyses also revealed a somewhat unexpected pattern: Recombination suppression can more often be driven by the fixation of homogametic (X-linked or Z-linked) rather than heterogametic (Y-linked or W-linked) modifiers. Such a pattern arises when recombination suppression is driven by sexual antagonism in populations where both sexes show similar fitness distributions. This is observed despite suppressors appearing on the Y or W chromosome exhibiting a stronger selective advantage than those appearing on the X or Z (Blackmon & Brandvain, 2017; Charlesworth & Charlesworth, 1980). This is because the difference in positive selection for these two types of suppressor is insufficient to offset the threefold discrepancy in their mutational input (owing to the homogametic chromosomes’ larger copy numbers).

This bias toward X-linked or Z-linked suppression is mild, but can be amplified for XY systems in taxa that show high male reproductive variance because selection is less effective for Y-linked mutations due to the Y’s reduced effective population size. In ZW systems, however, elevated male reproductive variance instead produces the reverse pattern: a strong W-bias in suppression, which is because such variance exacerbates drift on the Z-chromosome and does not influence the W. A bias toward X- or Z-linked modifiers is also observed when recombination arrest is solely due to the fixation of lucky inversions. The precise level of X/Z bias here is the outcome of multiple factors, including Muller’s ratchet acting on Y and W inversions (Roze & Lenormand, 2025), and the larger initial frequency of heterogametic mutations. By contrast, when recombination suppression evolves neutrally (Ironside, 2010), evolutionary rates on the X and Y will be equivalent because X–Y differences in the fixation probabilities of neutral alleles exactly cancel differences in mutational input, irrespective of sex-specific drift.

By showing that suppressors on the homogametic sex chromosome may be at least as influential as those on the heterogametic chromosome in driving recombination suppression, our results provide a potential explanation for the empirical observations of X- and Z-linked inversions along sex chromosomes in a wide range of taxa (e.g., in fish, plants, flies, and birds; Liu et al., 2025; McAllister, 2003; Moraga et al., 2025; Yue et al., 2023; Zhou et al., 2014). While current data remain too limited to draw firm conclusions (Liu et al., 2025), comparing the distribution of sex chromosome inversions across homo- and hetero-gametic chromosomes, especially in species with well-characterized mating systems, may offer a promising approach to delineate the roles of selective and non-selective processes in driving sex chromosome evolution. According to our findings, if such comparisons were to reveal a bias toward X-linked inversions in XY systems and W-linked inversions in ZW systems across species with high male reproductive variance, this would implicate sexual antagonism as driving sex chromosome evolution.

Our results can also provide insight into the relative rates of sex chromosome evolution in XY and ZW systems. In species with stronger male reproductive variance, we show that adaptive recombination suppression evolves more rapidly in ZW than XY systems (all else being equal), as efficient selection in females relative to males leads to higher substitution rates for suppressors capturing female-beneficial alleles on the W chromosome. Meanwhile, recombination suppression without sexual antagonism (i.e., driven by neutral or lucky inversions) should proceed equally quickly in XY and ZW systems. Because recombination arrest promotes genetic differentiation between the hetero- and homogametic sex chromosomes—often leading to W and Y degeneration (Bachtrog, 2013)—this would suggest that heteromorphic sex chromosomes may evolve more rapidly in systems with ZW than XY sex-determination when driven by sexual antagonism. Moreover, because sex chromosome divergence is thought to stabilize sex-determination systems in the face of the appearance of new sex-determining loci (Pokorna & Kratochvíl, 2009; Vicoso, 2019) (although see Blaser et al., 2013, 2014 for a contrasting theory), our results would suggest that ZW systems evolving with sexual antagonism may also turnover less frequently than XY systems in polygynous species. Taken together, these results generate predictions for how rates of recombination suppression evolution should differ among X and Y, Z and W, and XY and ZW systems depending on mating system and on whether regions of recombination suppression are influenced by sexual antagonism, deleterious variation, or genetic drift alone; we summarize these patterns in Table 1.

**Table 1.** Predictions for patterns of sex chromosome evolution under different drivers of recombination suppression.

Mating system	SA	SA and deleterious	Neutral	Deleterious
		variation		variation
Equal variance	$\mathrm{X}\gtrsim \mathrm{Y}$	$\mathrm{X}\gtrsim \mathrm{Y}$	$\mathrm{X}=\mathrm{Y}$	$\mathrm{X}\gtrsim \mathrm{Y}$
	$\mathrm{Z}\gtrsim \mathrm{W}$	$\mathrm{Z}\gtrsim \mathrm{W}$	$\mathrm{Z}= \mathrm{W}$	$\mathrm{Z}\gtrsim \mathrm{W}$
	$\mathrm{X}\mathrm{Y}=\mathrm{Z}\mathrm{W}$	$\mathrm{X}\mathrm{Y}=\mathrm{Z}\mathrm{W}$	$\mathrm{X}\mathrm{Y}=\mathrm{Z}\mathrm{W}$	$\mathrm{X}\mathrm{Y}=\mathrm{Z}\mathrm{W}$
Male-biased variance	$\mathrm{X} > \mathrm{Y}^\dagger$	$\mathrm{X} > \mathrm{Y}^\dagger$	$\mathrm{X}=\mathrm{Y}$	$\mathrm{X}\approx \mathrm{Y}$
	$\mathrm{Z} < \mathrm{W}^\dagger$	$\mathrm{Z} < \mathrm{W}^\dagger$	$\mathrm{Z}= \mathrm{W}$	$\mathrm{Z}\approx \mathrm{W}$
	$\mathrm{X}\mathrm{Y} < \mathrm{Z}\mathrm{W}^\dagger$	$\mathrm{X}\mathrm{Y} < \mathrm{Z}\mathrm{W}^\dagger$	$\mathrm{X}\mathrm{Y}=\mathrm{Z}\mathrm{W}$	$\mathrm{X}\mathrm{Y}=\mathrm{Z}\mathrm{W}$

*Note*. Predictions for the rate of recombination suppression when suppressors (i) capture a sexually antagonistic (SA) locus, (ii) capture sexually antagonistic (SA) and deleterious loci, (iii) are neutral, or (iv) capture deleterious loci, under scenarios in which male and female reproductive variance is either equal (as in the random-union-of-gametes model) or male-biased (as in our lottery polygyny model). Columns are ordered according to the overall pace of sex chromosome evolution predicted under each driver (pure sexual antagonism being fastest and pure deleterious variation slowest). In this table X vs. Y and Z vs. W denote the relative substitution rates of the X and Z chromosomes, respectively, while XY vs. ZW refers to the overall pace of recombination evolution in XY systems relative to ZW systems (by slight abuse of notation, > and < denote faster and slower recombination evolution, respectively, and $\gtrsim$ denotes faster or similar rates). Cells containing a $\dagger$ superscript are those with unique patterns for suppression driven by sexually antagonistic selection. All these baseline expectations are “all else equal,” i.e., assuming no differences in mutation rates, intrinsic costs of suppression, or strengths of selection between different mating systems, sex chromosomes, or sex-determination systems.

### Limitations and possible extensions

Our study considered explicitly cis-acting recombination modifiers (i.e., those that are in linkage to the SDR and trait loci) such as sex chromosome inversions (Kirkpatrick, 2010). One relevant factor of inversion biology that we did not consider is that these structural mutations can also induce fitness costs when present in heterozygous form, owing to the production of inviable gametes or zygotes (Berdan et al., 2023, for review). Importantly, if these costs act sex-specifically (which they may well do), then they could have a strong influence on the relative fitnesses of recombination suppressors on different sex chromosomes, and so on the balance of X(Z)-driven vs. Y(W)-driven recombination evolution. For example, gamete inviability may disproportionately harm females in many species (as sperm production is more weakly correlated with reproductive success than egg production unless males experience strong postcopulatory sexual selection, Janicke et al., 2016; Lehtonen, 2022), and so will penalize the invasion of X- or W-linked suppressors relative to those on the Y or Z. This effect could be sufficient to reverse the X- and W-bias in the basis of recombination suppression evolution. We therefore suggest that incorporating costs of suppression into recombination modifier models may be an important step toward understanding selection on inversions arising on different sex chromosomes.

In addition to modifiers acting in cis, recombination can be suppressed by trans-acting factors (Charlesworth, 2017; Jay et al., 2024) that are unlinked from the SDR and the trait locus (e.g., autosomal modifiers). In such cases, sex-specific selection can have diverse outcomes on recombination rates. In XY systems, selection on trans-acting modifiers can in fact favor the preservation, rather than suppression, of recombination on sex chromosomes. This occurs when males exhibit heterozygote advantage at a trait locus, but females show homozygote advantage for the allele that is at high frequency on the Y (Otto, 2014). However, we find that such a situation does not arise when allelic values diverge through gradual evolution, because the male-beneficial/female-deleterious allele is necessarily the one that increases in frequency on the Y (although it might arise if a large-effect female-beneficial mutation were introduced via male-biased introgression). Our results therefore suggest the evolution of allelic values in the PAR leads to polymorphism that favors recombination suppression by both cis- and trans-acting modifiers.

In this study, we have assumed that modifiers always suppress recombination, and we have focused on the rate at which such suppressors substitute in the population. It is possible, however, that mutations arise that undo recombination suppression once it has been established, namely chromosomal reversions. If (i) such reversions are sufficiently frequent and (ii) there are constraints on mechanisms that stabilize recombination suppression, such as dosage compensation (Lenormand & Roze, 2022, 2024), then reversions could slow sex chromosome evolution (Jay et al., 2024; Lenormand & Roze, 2024). Reversions would be especially consequential for neutral or lucky suppressors, because these modifiers are not maintained by selection once they have fixed. A reversion would therefore be neutral or even favored if restoring recombination improves the efficacy of selection against deleterious mutations accumulated on the non-recombining background through Hill–Robertson interference (Lenormand & Roze, 2024). This effect should be strongest for reversions on the heterogametic sex chromosome, where recombination arrest is complete, and interference is greatest. By contrast, the potential advantage of a reversion on the homogametic chromosome is less clear, because that chromosome continues to recombine and therefore experiences weaker interference. Instead, restoration of recombination could be favored if, after an inversion has fixed on the X/Z, a perfectly homologous inversion subsequently arises on the Y/W, re-establishing collinearity between the sex chromosomes. More generally, adaptive suppressors capturing sexually antagonistic loci should be more robust to suppression-reversing mutations, although reversions may still invade if the accumulation of deleterious variation on the non-recombining background becomes sufficiently strong. While the prevalence of reversions on sex chromosomes remains an open question, extending previous work (Lenormand & Roze, 2022, 2024) to examine their effects on rates of recombination suppression in the presence of both sexual antagonism and deleterious variation would help clarify their potential role in sex chromosome evolution.

## Conclusion

Sexually antagonistic selection has long been considered a key driver of sex chromosome evolution (Charlesworth & Charlesworth, 1980; Fisher, 1931; Ponnikas et al., 2018; Wright et al., 2016), favoring the capture of beneficial alleles by recombination suppressors and accelerating sex chromosome divergence. More recently, it has been suggested that recombination arrest may instead be driven by the fixation of neutral suppressors (Ironside, 2010; Jeffries et al., 2021) or “lucky” ones that capture fewer than expected deleterious mutations (Jay et al., 2024; Lenormand & Roze, 2022). These theories have been partly motivated by the difficulty of directly observing sexual antagonism driving recombination evolution (Jay et al., 2024; Lenormand & Roze, 2022). Our results show that recombination suppression due to sexual antagonism evolves on a much faster timescale than that for neutral or lucky suppressors. Moreover, we find this is true even when sexual antagonism is sufficiently weak that it would be challenging to detect empirically. Beyond this, our analyses reveal a poorly appreciated pathway for sex chromosome evolution: Recombination suppression may more often be driven by suppressors arising on the homogametic rather than the heterogametic chromosome. This bias should be especially pronounced in XY systems with high male reproductive variance, where selection on Y-linked suppressors shows diminished efficacy but selection on the X is largely unaffected. Testing these predictions empirically—especially in taxa where XY and ZW systems show different levels of recombination suppression—could help resolve the role of sexual antagonism in shaping sex chromosome evolution.

## Supplementary Material

qrag021_Supplemental_Files

## Data Availability

No new data were generated or analyzed in support of this research. Example SLiM simulation scripts and a Mathematica Notebook are included as supplementary files.
