# Supplementary material for: Sexual antagonism, mating systems, and recombination suppression on sex chromosomes
Source: Evol Lett. 2026 May 18;10(4):413–26. doi: 10.1093/evlett/qrag021 (PMC13451779; doi:10.1093/evlett/qrag021)
Supplement: Supplementary file 1 [file qrag021_supplemental_files.zip › PAR_SI.pdf]

Supplementary Figures

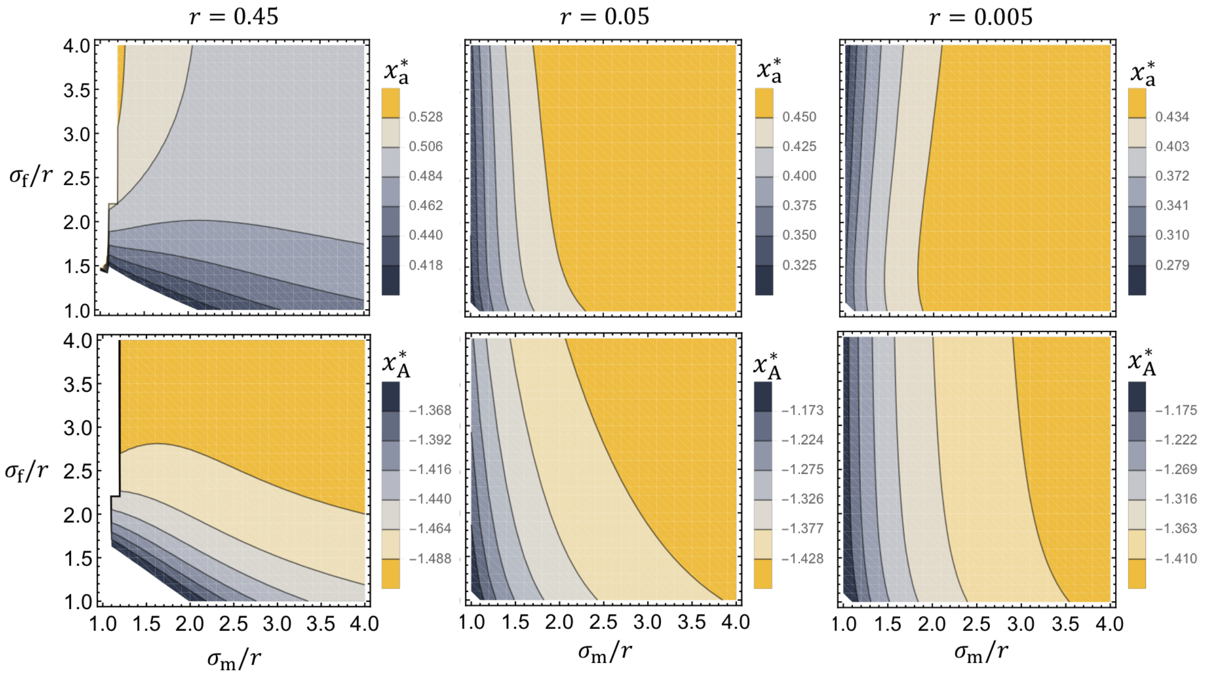

Supplementary Figure 1: **Allelic values at the evolutionary stable values (x_a^*, x_A^*).** Top row panels show the allelic value for x_a^* at the stable values (x_a^*, x_A^*) (Appendix A for calculation) as a function of the strength of male- and female-selection at the trait locus relative to the recombination rate (σ_m/r and σ_f/r). Bottom rows show equivalent results for the allelic value (x_A^*). Row panels show results for different strengths of recombination between the trait locus and SDR (r). Non-coloured space corresponds to where evolutionary branching at the trait locus does not occur (condition (A-2) not fulfilled). As seen under strictly symmetric sex-specific selection ($\sigma = \sigma_m = \sigma_f$, Fig. 1C), $x_a^* \approx 0.5$ and $x_A \approx -1.5$ unless sexually antagonistic selection is weak relative to the strength of recombination (σ_m and σ_f small while r big).

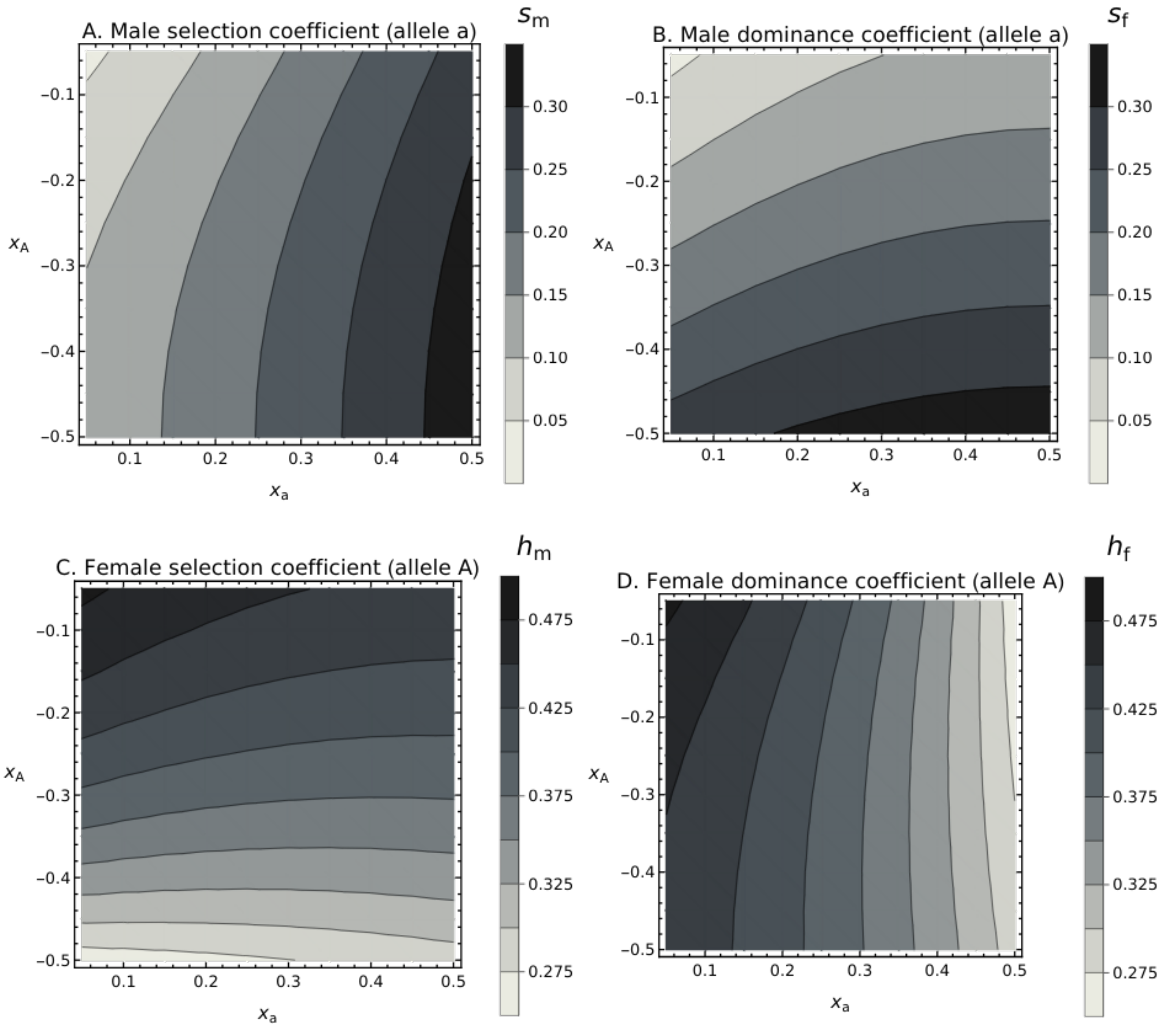

Supplementary Figure 2: **Selection and dominance coefficients in convention of Kidwell et al. (1977)**. Panels **A.** and **B.** show male (s_m) and female (s_f) selection coefficients, defined as the relative fitness cost when homozygote for the non-preferred allele: allele a imposes a cost $s_m = [w_m(2x_A) - w_m(2x_a)]/w_m(2x_A)$ in males, and alleles A imposes a cost $s_f = [w_f(2x_a) - w_f(2x_A)]/w_f(2x_a)$ in females. Panels **C.** and **D.** show the male (h_m) and female (h_f) dominance coefficients: $h_m = [w_m(2x_A) - w_m(x_a + x_A)]/[w_m(2x_A) - w_m(2x_a)]$ and $h_f = [w_f(2x_a) - w_f(x_a + x_A)]/[w_f(2x_a) - w_f(2x_A)]$. Values are plotted here for $\sigma = \sigma_m = \sigma_f = 0.1$ as in main text Fig. 2.

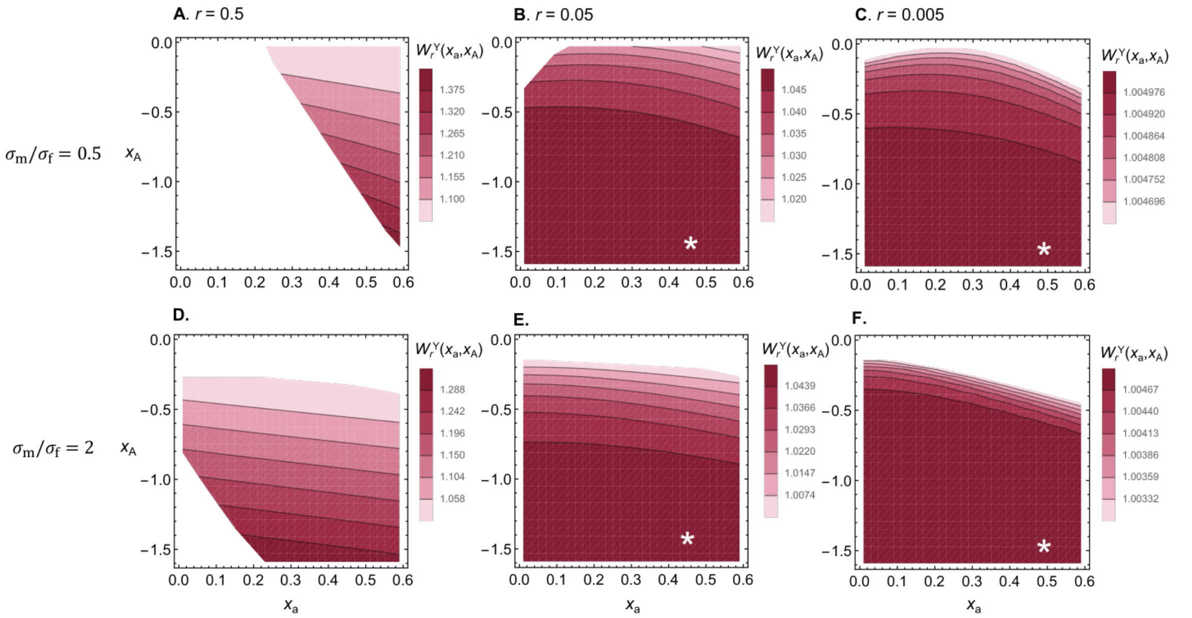

Supplementary Figure 3: **Invasion fitness of a Y-linked suppressor capturing a trait locus with alleles encoding x_a and x_A under sex-biased selection**. Plots show the invasion fitness, $W_r^\mathrm{Y}(x_\mathrm{a}, x_\mathrm{A})$ (main text eq. C-4, Appendix C.1 for calculation details), of a Y-linked recombination suppressor capturing allele A at the trait locus as a function of the allelic values x_a and x_A. Panels **A-C** (top row) show invasion fitness in the presence of female-biased selection at the trait locus ($\sigma_\mathrm{m} = 0.075, \sigma_\mathrm{f} = 0.15$, so that $\sigma_\mathrm{m}/\sigma_\mathrm{f} = 0.5$), and panels **D-F** show results for male-biased selection ($\sigma_\mathrm{m} = 0.15, \sigma_\mathrm{f} = 0.075$, so that $\sigma_\mathrm{m}/\sigma_\mathrm{f} = 2$). Different row panels show results for different levels of resident recombination between the trait locus and the SDR, r. White star shows the location of the evolutionary stable allelic values $(x_\mathrm{a}^*, x_\mathrm{A}^*)$. Compared to the case of equally strong selection in both sexes (see Fig. 2), the conditions for a Y-linked suppressor to invade are more restrictive under sex-biased selection, which is because such selection is less amenable to the maintenance of a balanced polymorphism at the trait locus (i.e., maintains polymorphism for a narrower range of allelic value combinations; x_a and x_A).

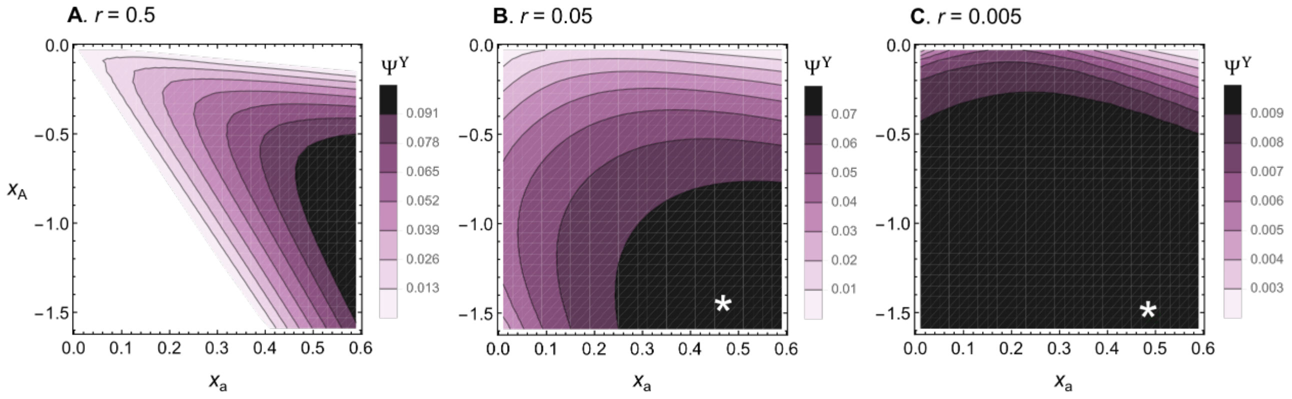

Supplementary Figure 4: **Invasion probability of a Y-linked suppressor capturing a trait locus with alleles encoding x_a and x_A.** Plots show the invasion probability under the random-union-of-gametes model, Ψ^Y (Appendix C.3.1 for calculation details), of a Y-linked recombination suppressor capturing allele A at the trait locus as a function of the allelic values x_a and x_A. Different panels show results for different levels of resident recombination between the trait locus and the SDR, r. White star shows the location of the evolutionary stable allelic values $(x_\mathrm{a}^*, x_\mathrm{A}^*)$. Other parameters used $\sigma = \sigma_\mathrm{m} = \sigma_\mathrm{f} = 0.1$. See SI Fig. 2 for the selection and dominance coefficients of alleles A and a in the notation of Kidwell et al. (1977).

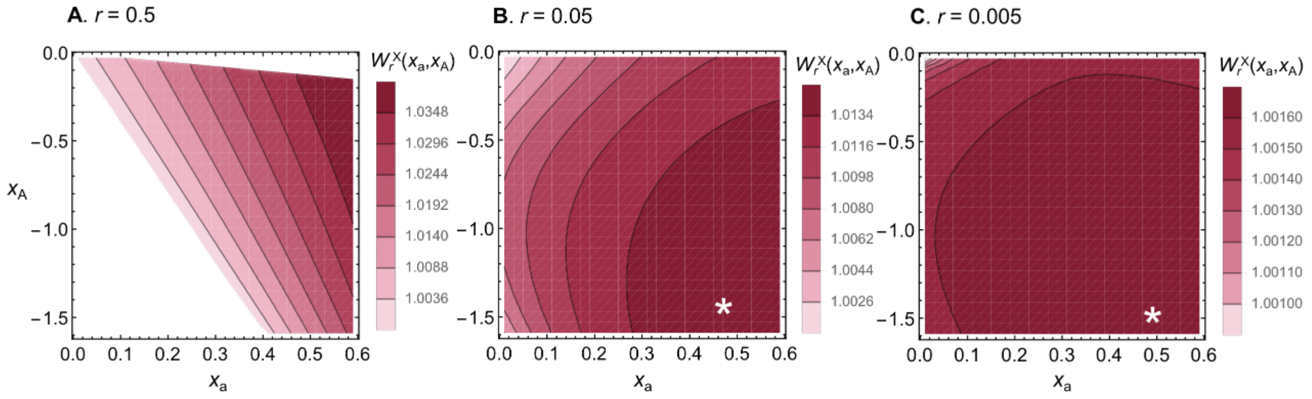

Supplementary Figure 5: **Invasion fitness of an X-linked suppressor capturing a trait locus with alleles encoding x_a and x_A.** Plots show the invasion fitness, $W_r^\mathrm{X}(x_\mathrm{a}, x_\mathrm{A})$ (main text eq. C-4, Appendix D.1 for calculation details), of an X-linked recombination suppressor capturing allele a at the trait locus as a function of the allelic values x_a and x_A. Different panels show results for different levels of resident recombination between the trait locus and the SDR, r. White stars shows the location of the evolutionary stable allelic values $(x_\mathrm{a}^*, x_\mathrm{A}^*)$. Other parameters used $\sigma = \sigma_\mathrm{m} = \sigma_\mathrm{f} = 0.1$. See SI Fig. 2 for the selection and dominance coefficients of alleles A and a in the notation of Kidwell et al. (1977).

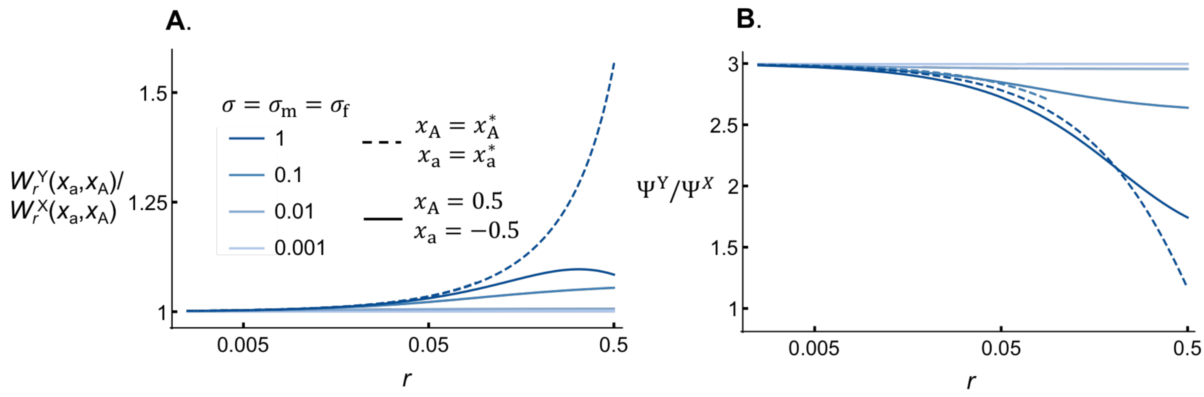

Supplementary Figure 6: **Invasion fitness and invasion probability for Y- vs X-linked suppressors**. Panel **A.** shows the ratio of the invasion fitnesses ($W_r^Y(x_a, x_A)/W_r^X(x_a, x_A)$) of a Y-linked suppressor capturing allele A at the trait locus and an X-linked suppressor capturing allele a for different recombination rates r and strength of sexually antagonistic selection $\sigma = \sigma_m = \sigma_f$. Dashed lines correspond to results for evolutionary stable allelic values (x_a^*, x_A^*) and solid lines for $x_a = 0.5, x_A = -0.5$ (Appendices C.1 and D.1 for calculation of $W_r^Y(x_a, x_A)$ and $W_r^X(x_a, x_A)$). Panel **B.** shows the ratio of the Y- vs X-linked invasion probabilities (Ψ^Y/Ψ^X) under the random-union-of-gametes model for the same parameter combinations and using the same legend as in panel A (Appendices C.3.1 and D.2.1 for calculation of Ψ^Y and Ψ^X).

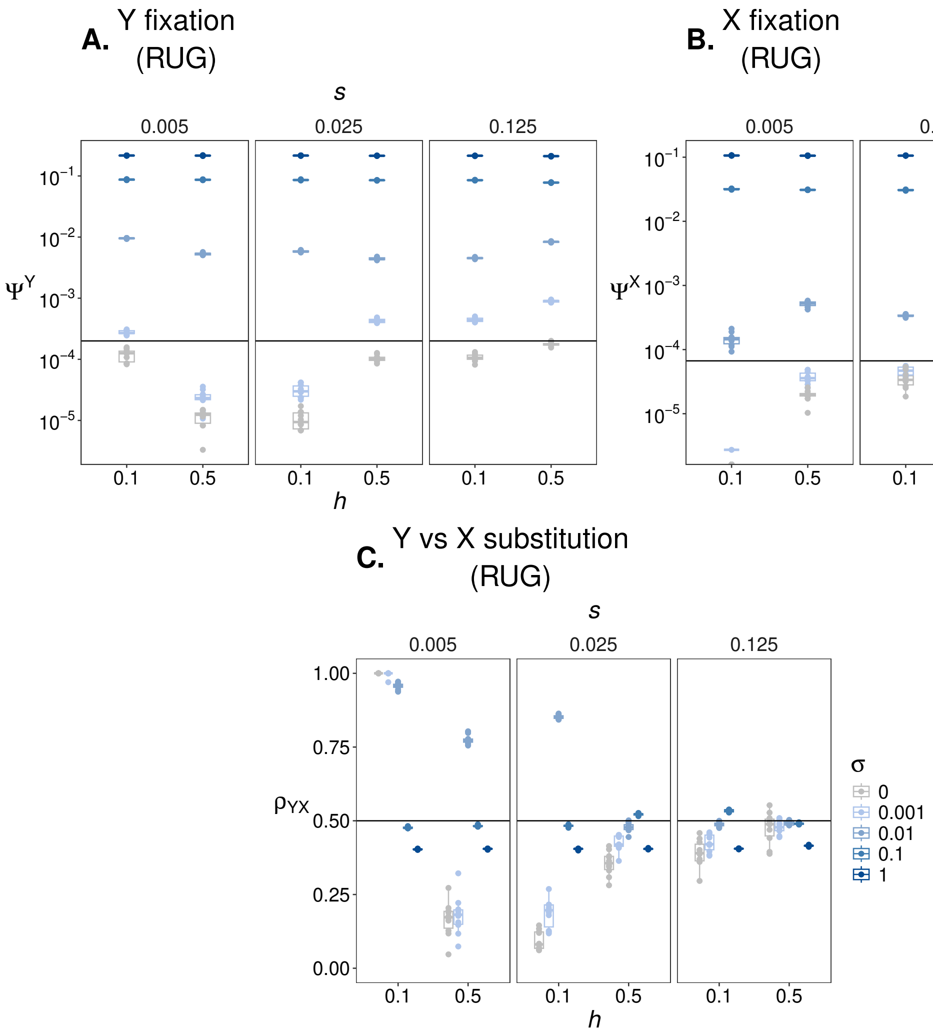

Supplementary Figure 7: **Selection for recombination suppression when deleterious and sexually antagonistic alleles co-segregate**. Panel **A.** shows the fixation probabilities estimated from individual-based-simulations of a Y-linked suppressor capturing both a sexually antagonistic locus and $L = 100$ deleterious loci at mutation-selection balance (Appendix E for simulation details). Results are shown for selection coefficient $s = 0.005$, $s = 0.025$, and $s = 0.125$ (see columns) and dominance coefficients of either $h = 0.1$ and $h = 0.5$ (see x-axis) at all deleterious loci. Each dot is an estimate for a single replicate population in a simulation of the random union-of-gametes model. Boxplot and dot colours correspond to different strengths of sexually antagonistic selection (σ). Horizontal line is the neutral fixation probability for a Y-linked mutation ($1/(N/2)$), where $N = 10^4$. Recombination is scaled such that $r = 0.3$ between the sexually antagonistic locus and SDR (Appendix E). Panel **B**. shows equivalent results as panel A, but for X-linked suppressors (here the neutral probability is $1/(3N/2)$). Panel **C.** shows the probability that a substitution by a recombination suppressor is Y- vs. X-linked, ρ_{YX}, from the same simulation setup as in panel A. Horizontal line marks $\rho_{\mathrm{YX}} = 0.5$. Here, XY and ZW systems are interchangeable: $\Psi^{\mathrm{Y}} = \Psi^{\mathrm{W}}$, $\Psi^{\mathrm{X}} = \Psi^{\mathrm{Z}}$, and $\rho_{\mathrm{YX}} = \rho_{\mathrm{WZ}}$.

Appendix to "Sexual antagonism, mating systems, and recombination suppression on sex chromosomes"

Ewan Flintham[1,*] and Charles Mullon[1]

[1]Department of Ecology and Evolution, University of Lausanne, 1015 Lausanne, Switzerland

[*]Correspondence: ewan.flintham@unil.ch

A Evolution of polymorphism on the PAR

In this appendix, we detail gradual evolution at the trait locus on the PAR under the continuum of alleles model.

A.1 Emergence of polymorphism

Under the model described in main text sections 2.1-2.2, gradual evolution at a locus under sex-specific selection has been studied with general fitness functions $w_{\mathrm{f}}(z)$ and $w_{\mathrm{m}}(z)$ in Flintham et al. (2025). They showed that selection first drives the population towards an equilibrium at $z^* = 2x^*$. With Gaussian fitness ($w_{\mathrm{m}}(z) = e^{-\sigma_{\mathrm{m}}(1+z)^2}$ and $w_{\mathrm{f}}(z) = e^{-\sigma_{\mathrm{f}}(-1+z)^2}$), we can find this equilibrium by plugging $w_{\mathrm{m}}(z)$ and $w_{\mathrm{f}}(z)$ into eq. (A-11) in Flintham et al. (2025) and solving for z^*. This readily gives

$$z^* = 2x^* = \frac{\sigma_{\mathrm{f}} - \sigma_{\mathrm{m}}}{\sigma_{\mathrm{f}} + \sigma_{\mathrm{m}}}, \tag{A-1}$$

(it is also straightforward to see that this equilibrium is always convergence stable, i.e., is an attractor of evolutionary dynamics, by plugging in $w_{\mathrm{m}}(z)$ and $w_{\mathrm{f}}(z)$ into their eq. A-12).

Once the population expresses z^*, selection is then either stabilising, such that allelic values remain distributed around x^* under mutation-selection balance; or disruptive whereby their distribution

becomes bimodal in a process referred to as "evolutionary branching" leading to the maintenance of a polymorphism under balancing selection (Dercole and Rinaldi, 2008; Geritz et al., 1998; Rueffler et al., 2006). The condition for disruptive selection is found by plugging Gaussian $w_{\mathrm{m}}(z)$ and $w_{\mathrm{f}}(z)$ into eq. (E-27) of Flintham et al. (2025), which after some re-arrangements reads

$$r < \frac{8\sigma_{\mathrm{m}}^2\sigma_{\mathrm{f}}^2}{(\sigma_{\mathrm{m}}+\sigma_{\mathrm{f}})^3} \tag{A-2}$$

(see eq. B-25 in Flintham et al., 2025 for condition with general male and female fitness functions). When selection is of equal strength in males and females, $\sigma = \sigma_{\mathrm{m}} = \sigma_{\mathrm{f}}$, this condition simplifies to $r < \sigma$.

If condition (A-2) holds, the population will undergo evolutionary branching, leading to the emergence of two alleles (denoted a and A) that are held in a balanced polymorphism and that undergo further divergence (Geritz et al., 1998). We characterise this second stage of evolution in the following section.

A.2 Divergence and maintenance polymorphism

The evolution of allelic values for a and A can be investigated through an invasion analysis that is based on the invasion fitness $W(x_\bullet, x_{\mathrm{a}}, x_{\mathrm{A}})$ of a rare mutant encoding an allelic value $x_\bullet$ in a population where resident A and a alleles – encoding x_{a} and x_{A}, respectively – are at their equilibrium frequencies (Dercole and Rinaldi, 2008; Geritz et al., 1998; Otto and Day, 2011, chapter 12). The direction of the evolution of allelic effects a and A is determined by the selection gradient on each genetic value: x_{A} will evolve according to the sign of $s_{\mathrm{A}}(x_{\mathrm{A}}, x_{\mathrm{a}}) = \partial W(x_\bullet, x_{\mathrm{A}}, x_{\mathrm{a}})/\partial x_\bullet|_{x_\bullet = x_{\mathrm{A}}}$ and x_{a} to the sign of $s_{\mathrm{a}}(x_{\mathrm{A}}, x_{\mathrm{a}}) = \partial W(x_\bullet, x_{\mathrm{A}}, x_{\mathrm{a}})/\partial x_\bullet|_{x_\bullet = x_{\mathrm{a}}}$. Such evolution may therefore halt at an equilibrium allelic pair of values (x_{A}^*, x_{a}^*) or "coalition" (Brown and Vincent, 1987) that satisfies

$$s_{\mathrm{A}}(x_{\mathrm{A}}^*, x_{\mathrm{a}}^*) = s_{\mathrm{a}}(x_{\mathrm{A}}^*, x_{\mathrm{a}}^*) = 0. \tag{A-3}$$

This coalition is convergence stable if the real part of the leading eigenvalue of the Jacobian matrix

$$\mathrm{J}(x_\mathrm{a}^*, x_\mathrm{A}^*) = \begin{pmatrix} \dfrac{\partial s_\mathrm{a}(x_\mathrm{A}, x_\mathrm{a})}{\partial x_\mathrm{a}} & \dfrac{\partial s_\mathrm{a}(x_\mathrm{A}, x_\mathrm{a})}{\partial x_\mathrm{A}} \\ \\ \dfrac{\partial s_\mathrm{A}(x_\mathrm{A}, x_\mathrm{a})}{\partial x_\mathrm{a}} & \dfrac{\partial s_\mathrm{A}(x_\mathrm{A}, x_\mathrm{a})}{\partial x_\mathrm{A}} \end{pmatrix}_{\substack{x_\mathrm{a}=x_\mathrm{a}^* \\ x_\mathrm{A}=x_\mathrm{A}^*}} \tag{A-4}$$

is negative, and is evolutionarily stable, i.e., there is no further branching once allelic values have converged to x_a^* and x_A^*, if

$$\begin{aligned} \left.\frac{\partial^2 W(x_\bullet, x_\mathrm{A}, x_\mathrm{a})}{\partial x_\bullet^2}\right|_{x_\bullet = x_\mathrm{a} = x_\mathrm{a}^*} &< 0 \\ \left.\frac{\partial^2 W(x_\bullet, x_\mathrm{A}, x_\mathrm{a})}{\partial x_\bullet^2}\right|_{x_\bullet = x_\mathrm{A} = x_\mathrm{A}^*} &< 0, \end{aligned} \tag{A-5}$$

in which case the coalition $(x_\mathrm{a}^*, x_\mathrm{A}^*)$ is an endpoint to gradual evolution.

A.2.1 Invasion fitness

We now characterise the invasion fitness $W(x_\bullet, x_\mathrm{A}, x_\mathrm{a})$ of a mutant encoding an allelic value $x_\bullet$ for our model. A mutant allele may initially appear in one of three chromosomal settings: it may arise (i) in an (necessarily X-bearing) egg cell, (ii) in an X-bearing sperm cell, and (iii) in a Y-bearing sperm cell. Its invasion fitness $W(x_\bullet, x_\mathrm{a}, x_\mathrm{A})$ is therefore given by the leading eigenvalue of the mean matrix

$$\mathrm{M}(x_\bullet, x_\mathrm{a}, x_\mathrm{A}) = \begin{pmatrix} W^{\mathrm{f}\to\mathrm{f}}(x_\bullet, x_\mathrm{a}, x_\mathrm{A}) & W^{\mathrm{mX}\to\mathrm{f}}(x_\bullet, x_\mathrm{a}, x_\mathrm{A}) & W^{\mathrm{mY}\to\mathrm{f}}(x_\bullet, x_\mathrm{a}, x_\mathrm{A}) \\ \\ W^{\mathrm{f}\to\mathrm{mX}}(x_\bullet, x_\mathrm{a}, x_\mathrm{A}) & W^{\mathrm{mX}\to\mathrm{mX}}(x_\bullet, x_\mathrm{a}, x_\mathrm{A}) & W^{\mathrm{mY}\to\mathrm{mX}}(x_\bullet, x_\mathrm{a}, x_\mathrm{A}) \\ \\ W^{\mathrm{f}\to\mathrm{mY}}(x_\bullet, x_\mathrm{a}, x_\mathrm{A}) & W^{\mathrm{mX}\to\mathrm{mY}}(x_\bullet, x_\mathrm{a}, x_\mathrm{A}) & W^{\mathrm{mY}\to\mathrm{mY}}(x_\bullet, x_\mathrm{a}, x_\mathrm{A}) \end{pmatrix} \tag{A-6}$$

where $W^{u\to v}(x_\bullet, x_\mathrm{a}, x_\mathrm{A})$ is the expected number of recruited mutant gametes (i.e., that will go on to successfully form a surviving adult individual) of type $v \in \{\mathrm{f}, \mathrm{mX}, \mathrm{mY}\}$ at generation $t+1$ produced by a recruited mutant gamete of type $u \in \{\mathrm{f}, \mathrm{mX}, \mathrm{mY}\}$ at generation t (where superscript f denotes presence in eggs, mX in X-bearing sperm, mY in Y-bearing sperm).

It is straightforward to show that according to the life cycle of the population, the individual components of invasion fitness in eq. (A-6) are

$$\begin{aligned}
W^{\mathrm{f}\to\mathrm{f}}(x_\bullet, x_{\mathrm{A}}, x_{\mathrm{a}}) &= \frac{1}{2}\left(\frac{w_{\mathrm{f}}(x_\bullet + x_{\mathrm{a}})}{\overline{w}_{\mathrm{f}}}\left(1-\widehat{p}^{\mathrm{X}}_{\mathrm{m}}\right) + \frac{w_{\mathrm{f}}(x_\bullet + x_{\mathrm{A}})}{\overline{w}_{\mathrm{f}}}\widehat{p}^{\mathrm{X}}_{\mathrm{m}}\right)\\
W^{\mathrm{mX}\to\mathrm{f}}(x_\bullet, x_{\mathrm{A}}, x_{\mathrm{a}}) &= \frac{1}{2}\left(\frac{w_{\mathrm{f}}(x_\bullet + x_{\mathrm{a}})}{\overline{w}_{\mathrm{f}}}\left(1-\widehat{p}_{\mathrm{f}}\right) + \frac{w_{\mathrm{f}}(x_\bullet + x_{\mathrm{A}})}{\overline{w}_{\mathrm{f}}}\widehat{p}_{\mathrm{f}}\right)\\
W^{\mathrm{f}\to\mathrm{mX}}(x_\bullet, x_{\mathrm{A}}, x_{\mathrm{a}}) &= (1-r)\left(\frac{w_{\mathrm{m}}(x_\bullet + x_{\mathrm{a}})}{\overline{w}_{\mathrm{m}}}\left(1-\widehat{p}^{\mathrm{Y}}_{\mathrm{m}}\right) + \frac{w_{\mathrm{m}}(x_\bullet + x_{\mathrm{A}})}{\overline{w}_{\mathrm{m},t}}\widehat{p}^{\mathrm{Y}}_{\mathrm{m}}\right)\\
W^{\mathrm{mY}\to\mathrm{mX}}(x_\bullet, x_{\mathrm{A}}, x_{\mathrm{a}}) &= r\left(\frac{w_{\mathrm{m}}(x_\bullet + x_{\mathrm{a}})}{\overline{w}_{\mathrm{m}}}\left(1-\widehat{p}_{\mathrm{f}}\right) + \frac{w_{\mathrm{f}}(x_\bullet + x_{\mathrm{A}})}{\overline{w}_{\mathrm{m}}}\widehat{p}_{\mathrm{f}}\right)\\
W^{\mathrm{f}\to\mathrm{mY}}(x_\bullet, x_{\mathrm{A}}, x_{\mathrm{a}}) &= r\left(\frac{w_{\mathrm{m}}(x_\bullet + x_{\mathrm{a}})}{\overline{w}_{\mathrm{m}}}\left(1-\widehat{p}^{\mathrm{Y}}_{\mathrm{m}}\right) + \frac{w_{\mathrm{m}}(x_\bullet + x_{\mathrm{A}})}{\overline{w}_{\mathrm{m}}}\widehat{p}^{\mathrm{Y}}_{\mathrm{m}}\right)\\
W^{\mathrm{mY}\to\mathrm{mY}}(x_\bullet, x_{\mathrm{A}}, x_{\mathrm{a}}) &= (1-r)\left(\frac{w_{\mathrm{m}}(x_\bullet + x_{\mathrm{a}})}{\overline{w}_{\mathrm{m}}}\left(1-\widehat{p}_{\mathrm{f}}\right) + \frac{w_{\mathrm{m}}(x_\bullet + x_{\mathrm{A}})}{\overline{w}_{\mathrm{m}}}\widehat{p}_{\mathrm{f}}\right),\\
W^{\mathrm{mY}\to\mathrm{f}}(x_\bullet, x_{\mathrm{A}}, x_{\mathrm{a}}) &= W^{\mathrm{mX}\to\mathrm{mY}}(x_\bullet, x_{\mathrm{A}}, x_{\mathrm{a}}) = W^{\mathrm{mX}\to\mathrm{mX}}(x_\bullet, x_{\mathrm{A}}, x_{\mathrm{a}}) = 0
\end{aligned} \tag{A-7}$$

where $\widehat{p}_{\mathrm{f}}$, $\widehat{p}^{\mathrm{X}}_{\mathrm{m}}$, and $\widehat{p}^{\mathrm{Y}}_{\mathrm{m}}$ are the equilibrium frequencies of A in eggs, in X-bearing sperm, and Y-bearing sperm, respectively, and

$$\begin{aligned}
\overline{w}_{\mathrm{f}} =& \widehat{p}^{\mathrm{X}}_{\mathrm{f}}\widehat{p}_{\mathrm{f}} w_{\mathrm{f}}(2x_{\mathrm{A}}) + \left(\widehat{p}^{\mathrm{X}}_{\mathrm{m}}(1-\widehat{p}_{\mathrm{f}}) + (1-\widehat{p}^{\mathrm{X}}_{\mathrm{m}})\widehat{p}_{\mathrm{f}}\right) w_{\mathrm{f}}(x_{\mathrm{A}}+x_{\mathrm{a}}) + (1-\widehat{p}^{\mathrm{X}}_{\mathrm{m}})(1-\widehat{p}_{\mathrm{f}})\, w_{\mathrm{f}}(2x_{\mathrm{a}})\\
\overline{w}_{\mathrm{m}} =& \widehat{p}^{\mathrm{Y}}_{\mathrm{m}}\widehat{p}_{\mathrm{f}} w_{\mathrm{m}}(2x_{\mathrm{A}}) + \left(\widehat{p}^{\mathrm{Y}}_{\mathrm{m}}(1-\widehat{p}_{\mathrm{f}}) + (1-\widehat{p}^{\mathrm{Y}}_{\mathrm{m}})\widehat{p}_{\mathrm{f}}\right) w_{\mathrm{m}}(x_{\mathrm{A}}+x_{\mathrm{a}}) + (1-\widehat{p}_{\mathrm{m}}\mathrm{Y})(1-\widehat{p}_{\mathrm{f}})\, w_{\mathrm{m}}(2x_{\mathrm{a}})
\end{aligned} \tag{A-8}$$

are mean female and male fitness when alleles A and a are at their equilibrium frequencies.

To find the equilibrium frequencies $\widehat{p}_{\mathrm{f}}$, $\widehat{p}^{\mathrm{X}}_{\mathrm{m}}$, and $\widehat{p}^{\mathrm{Y}}_{\mathrm{m}}$, we write the frequency of allele A in each chromosome at generation at $t+1$ as

$$\begin{aligned}
p^{\mathrm{X}}_{\mathrm{m},t+1} &= M^{\mathrm{X}}(p^{\mathrm{X}}_{\mathrm{m},t}, p^{\mathrm{Y}}_{\mathrm{m},t}, p_{\mathrm{f},t})\\
p^{\mathrm{Y}}_{\mathrm{m},t+1} &= M^{\mathrm{Y}}(p^{\mathrm{X}}_{\mathrm{m},t}, p^{\mathrm{Y}}_{\mathrm{m},t}, p_{\mathrm{f},t})\\
p_{\mathrm{f},t+1} &= F(p^{\mathrm{X}}_{\mathrm{m},t}, p^{\mathrm{Y}}_{\mathrm{m},t}, p_{\mathrm{f},t}),
\end{aligned} \tag{A-9}$$

where

$$\begin{aligned}
M^{\mathrm{X}}(p^{\mathrm{X}}_{\mathrm{m},t}, p^{\mathrm{Y}}_{\mathrm{m},t}, p_{\mathrm{f},t}) &= p^{\mathrm{Y}}_{\mathrm{m},t}p_{\mathrm{f},t}\frac{w_{\mathrm{m}}(2x_{\mathrm{A}})}{\overline{w}_{\mathrm{m},t}} + \left(p^{\mathrm{Y}}_{\mathrm{m},t}(1-p_{\mathrm{f},t})r + (1-p^{\mathrm{Y}}_{\mathrm{m},t})p_{\mathrm{f},t}(1-r)\right)\frac{w_{\mathrm{m}}(x_{\mathrm{A}}+x_{\mathrm{a}})}{\overline{w}_{\mathrm{m},t}}\\
M^{\mathrm{Y}}(p^{\mathrm{X}}_{\mathrm{m},t}, p^{\mathrm{Y}}_{\mathrm{m},t}, p_{\mathrm{f},t}) &= p^{\mathrm{Y}}_{\mathrm{m},t}p_{\mathrm{f},t}\frac{w_{\mathrm{m}}(2x_{\mathrm{A}})}{\overline{w}_{\mathrm{m},t}} + \left(p^{\mathrm{Y}}_{\mathrm{m},t}(1-p_{\mathrm{f},t})(1-r) + (1-p^{\mathrm{Y}}_{\mathrm{m},t})p_{\mathrm{f},t}r\right)\frac{w_{\mathrm{m}}(x_{\mathrm{A}}+x_{\mathrm{a}})}{\overline{w}_{\mathrm{m},t}}\\
F(p^{\mathrm{X}}_{\mathrm{m},t}, p^{\mathrm{Y}}_{\mathrm{m},t}, p_{\mathrm{f},t}) &= p^{\mathrm{X}}_{\mathrm{m},t}p_{\mathrm{f},t}\frac{w_{\mathrm{f}}(2x_{\mathrm{A}})}{\overline{w}_{\mathrm{f},t}} + \frac{1}{2}\left(p^{\mathrm{X}}_{\mathrm{m},t}(1-p_{\mathrm{f},t}) + (1-p^{\mathrm{X}}_{\mathrm{m},t})p_{\mathrm{f},t}\right)\frac{w_{\mathrm{f}}(x_{\mathrm{a}}+x_{\mathrm{A}})}{\overline{w}_{\mathrm{f},t}},
\end{aligned} \tag{A-10}$$

and

$$\overline{w}_{\mathrm{f},t} = p^{\mathrm{X}}_{\mathrm{f},t} p_{\mathrm{f},t} w_{\mathrm{f}}(2x_{\mathrm{A}}) + \left(p^{\mathrm{X}}_{\mathrm{m},t}(1-p_{\mathrm{f},t}) + (1-p^{\mathrm{X}}_{\mathrm{m},t})p_{\mathrm{f},t}\right) w_{\mathrm{f}}(x_{\mathrm{A}}+x_{\mathrm{a}}) + (1-p^{\mathrm{X}}_{\mathrm{m},t})(1-p_{\mathrm{f},t}) w_{\mathrm{f}}(2x_{\mathrm{a}})$$
$$\overline{w}_{\mathrm{m},t} = p^{\mathrm{Y}}_{\mathrm{m},t} p_{\mathrm{f},t} w_{\mathrm{m}}(2x_{\mathrm{A}}) + \left(p^{\mathrm{Y}}_{\mathrm{m},t}(1-p_{\mathrm{f},t}) + (1-p^{\mathrm{Y}}_{\mathrm{m},t})p_{\mathrm{f},t}\right) w_{\mathrm{m}}(x_{\mathrm{A}}+x_{\mathrm{a}}) + (1-p^{\mathrm{Y}}_{\mathrm{m},t})(1-p_{\mathrm{f},t}) w_{\mathrm{m}}(2x_{\mathrm{a}})$$
(A-11)

(Clark, 1988). Plugging eq. (A-10) into eq. (A-9), we can solve simultaneously for $\widehat{p}_{\mathrm{f}} = p_{\mathrm{f},t+1} = p_{\mathrm{f},t}$, $\widehat{p}^{\mathrm{X}}_{\mathrm{m}} = p^{\mathrm{X}}_{\mathrm{m},t+1} = p^{\mathrm{X}}_{\mathrm{m},t}$ and $\widehat{p}^{\mathrm{Y}}_{\mathrm{m}} = p^{\mathrm{Y}}_{\mathrm{m},t+1} = p^{\mathrm{Y}}_{\mathrm{m},t}$.

A.2.2 Numerical approach

Owing to the complexity of the system eq. (A-9)-(A-11), we could not find analytical expressions for $\widehat{p}_{\mathrm{f}}, \widehat{p}^{\mathrm{X}}_{\mathrm{m}}$ and $\widehat{p}^{\mathrm{Y}}_{\mathrm{m}}$. We therefore proceeded to find x^*_{A} and x^*_{a} with a numerical analysis assuming Gaussian fitness functions for $w_{\mathrm{m}}(z)$ and $w_{\mathrm{f}}(z)$. To do so, we iterated the recursions

$$x_{\mathrm{a},i+1} = x_{\mathrm{a},i} + \delta s_{\mathrm{a}}(x_{\mathrm{A},i}, x_{\mathrm{a},i})$$
$$x_{\mathrm{A},i+1} = x_{\mathrm{A},i} + \delta s_{\mathrm{A}}(x_{\mathrm{A},i}, x_{\mathrm{a},i}) \qquad \text{(A-12)}$$

with $\delta = 0.1/((\sigma_{\mathrm{m}} + \sigma_{\mathrm{f}})/2)$ (this scaling by σ_{m} and σ_{f} is made for computational expediency, so that changes in allelic values between iterations are not vanishingly small when selection is weak) until we reached a pair $(x_{\mathrm{A},i}, x_{\mathrm{a},i})$ that satisfied $|x_{\mathrm{A},i+1} - x_{\mathrm{A},i}| < 10^{-6}$ and $|x_{\mathrm{a},i+1} - x_{\mathrm{a},i}| < 10^{-6}$, which we took to be the equilibrium, i.e. $(x_{\mathrm{A},i}, x_{\mathrm{a},i}) = (x^*_{\mathrm{A}}, x^*_{\mathrm{a}})$. To calculate $s_{\mathrm{a}}(x_{\mathrm{A},i}, x_{\mathrm{a},i})$ and $s_{\mathrm{A}}(x_{\mathrm{A},i}, x_{\mathrm{a},i})$ at each iteration, we plugged $x_{\mathrm{a},i}$ and $x_{\mathrm{A},i}$ into eqs. (A-10) and eq. (A-11) to find the equilibrium frequencies $\widehat{p}_{\mathrm{f}}, \widehat{p}^{\mathrm{X}}_{\mathrm{m}}$ and $\widehat{p}^{\mathrm{Y}}_{\mathrm{m}}$, then plugged these allelic values and their associated equilibrium frequencies into eq. (A-7), then eq. (A-7) into eq. (A-6), from which we can calculate $s_{\mathrm{a}}(x_{\mathrm{a},i}, x_{\mathrm{A},i})$ and $s_{\mathrm{A}}(x_{\mathrm{a},i}, x_{\mathrm{A},i})$. Finally, we checked that the equilibrium $(x^*_{\mathrm{a}}, x^*_{\mathrm{A}})$ was convergence and evolutionarily stable by plugging these values into eqs. (A-4)-(A-5), which we found to be satisfied in all cases, indicating that x^*_{a} and x^*_{A} are stable endpoints to gradual evolution. We repeated this procedure with different parameter values (σ_{m}, σ_{f}, and r) to produce Fig 1C and SI Fig. 1. In each case, we initialised from a pair of weakly diverged allelic values ($x_{\mathrm{a},1} = 0.01, x_{\mathrm{A},1} = -0.01$).

A.3 Balanced polymorphism in the PAR

In addition to the evolutionary stable values $(x_{\mathrm{a}}^*, x_{\mathrm{A}}^*)$, we also investigate arbitrary allelic values x_{a} and x_{A} that lead to a balanced polymorphism at the trait locus. For such a polymorphism for two alleles encoding x_{a} and x_{A} to exist requires that at least one eigenvalue of the Jacobian matrix

$$\mathrm{J}(x_{\mathrm{a}}, x_{\mathrm{A}}) = \begin{pmatrix} \dfrac{\partial M^{\mathrm{X}}(p_{\mathrm{m},t}^{\mathrm{X}}, p_{\mathrm{m},t}^{\mathrm{Y}}, p_{\mathrm{f},t})}{\partial p_{\mathrm{m},t}^{\mathrm{X}}} & \dfrac{\partial M^{\mathrm{X}}(p_{\mathrm{m},t}^{\mathrm{X}}, p_{\mathrm{m},t}^{\mathrm{Y}}, p_{\mathrm{f},t})}{\partial p_{\mathrm{m},t}^{\mathrm{Y}}} & \dfrac{\partial M^{\mathrm{X}}(p_{\mathrm{m},t}^{\mathrm{X}}, p_{\mathrm{m},t}^{\mathrm{Y}}, p_{\mathrm{f},t})}{\partial p_{\mathrm{f},t}} \\ \dfrac{\partial M^{\mathrm{Y}}(p_{\mathrm{m},t}^{\mathrm{X}}, p_{\mathrm{m},t}^{\mathrm{Y}}, p_{\mathrm{f},t})}{\partial p_{\mathrm{m},t}^{\mathrm{X}}} & \dfrac{\partial M^{\mathrm{Y}}(p_{\mathrm{m},t}^{\mathrm{X}}, p_{\mathrm{m},t}^{\mathrm{Y}}, p_{\mathrm{f},t})}{\partial p_{\mathrm{m},t}^{\mathrm{Y}}} & \dfrac{\partial M^{\mathrm{Y}}(p_{\mathrm{m},t}^{\mathrm{X}}, p_{\mathrm{m},t}^{\mathrm{Y}}, p_{\mathrm{f},t})}{\partial p_{\mathrm{f},t}} \\ \dfrac{\partial F(p_{\mathrm{m},t}^{\mathrm{X}}, p_{\mathrm{m},t}^{\mathrm{Y}}, p_{\mathrm{f},t})}{\partial p_{\mathrm{m},t}^{\mathrm{X}}} & \dfrac{\partial F(p_{\mathrm{m},t}^{\mathrm{X}}, p_{\mathrm{m},t}^{\mathrm{Y}}, p_{\mathrm{f},t})}{\partial p_{\mathrm{m},t}^{\mathrm{Y}}} & \dfrac{\partial F(p_{\mathrm{m},t}^{\mathrm{X}}, p_{\mathrm{m},t}^{\mathrm{Y}}, p_{\mathrm{f},t})}{\partial p_{\mathrm{f},t}} \end{pmatrix}_{p_{\mathrm{m},t}^{\mathrm{X}}=p_{\mathrm{m},t}^{\mathrm{Y}}=p_{\mathrm{f},t}=p} \tag{A-13}$$

is greater than one in absolute value for both $p = 0$ and $p = 1$ (which indicates reciprocal invasion of alleles A and a), where the functions $M^{\mathrm{X}}(p_{\mathrm{m},t}^{\mathrm{Y}}, p_{\mathrm{m},t}^{\mathrm{Y}}, p_{\mathrm{f},t})$, $M^{\mathrm{Y}}(p_{\mathrm{m},t}^{\mathrm{Y}}, p_{\mathrm{m},t}^{\mathrm{Y}}, p_{\mathrm{f},t})$ and $F(p_{\mathrm{m},t}^{\mathrm{Y}}, p_{\mathrm{m},t}^{\mathrm{Y}}, p_{\mathrm{f},t})$ are detailed in eq. (A-10).

B Simulating polygenic variation on the PAR

In this Appendix, we investigate whether male heterozygote advantage evolves in response to sexually antagonistic selection (as found under the continuum-of-alleles model, Appendix A) when z shows a polygenic basis on the PAR. To do this, we ran simulations where z was encoded by 10 di-allelic quantitative trait loci (with additive effects on the phenotypes) under the random-union-of-gametes model (Appendix C.3.1 for implementation of this lifecycle in a simulation). At each locus one segregating allele encodes an effect 0.25 and the other −0.25; these values were chosen for illustrative purposes, as they allow for a wide range of haplotype values, including the common evolutionarily stable values in the continuum-of-alleles-model ($x_{\mathrm{a}}^* \approx 0.5, x_{\mathrm{A}}^* \approx -1.5$). We assumed loci were equally spaced along the PAR with a uniform recombination rate r (i.e., r is the probability of recombination between adjacent trait loci, and between the SDR and the closest terminal trait locus) and we initialised simulations with equal frequencies of each allele at each locus. We tracked the evolution of the genetic values encoded by X and Y chromosomes over 15000 generations of sexually antagonistic selection (under Gaussian fecundity functions with $\sigma = 0.1$ for illustration). Results are shown in

Appendix Fig. 1.

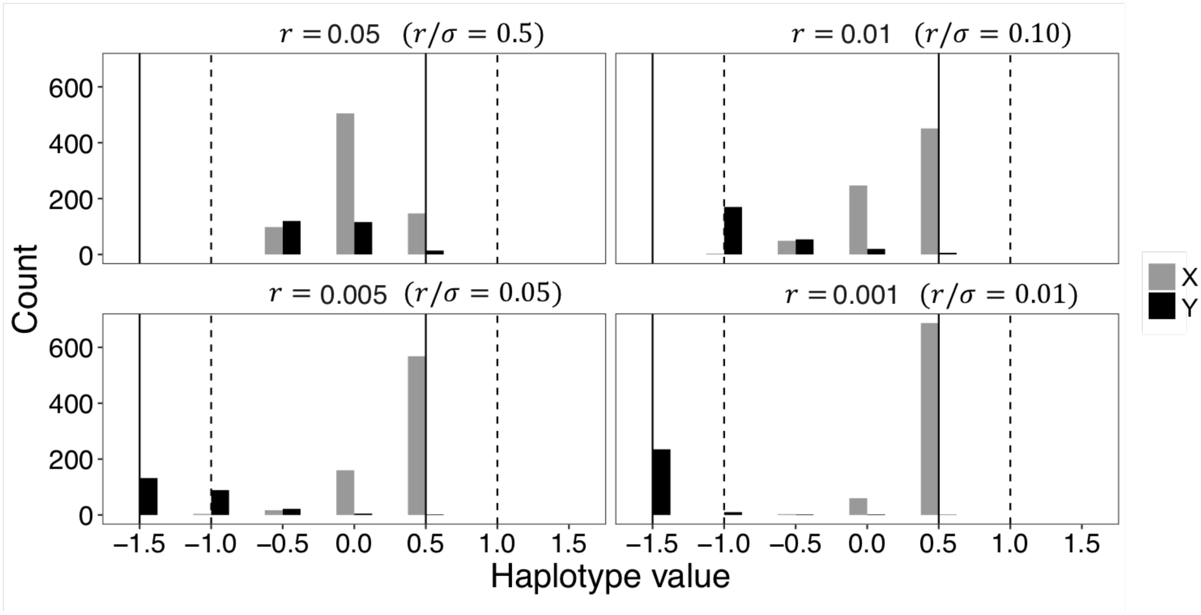

Appendix Figure 1: **Evolved haplotypes when z is polygenic**. Panels show distributions of genetic effects encoded by a sample of 1000 chromosomes (grey and black columns for X and Y chromosomes, respectively) segregating in the population after 15000 generations of a simulation. Different panels show results for different strengths of recombination amongst loci, r. Vertical dotted lines indicate the location of the male and female optima (at −1 and 1, respectively) and vertical solid lines indicate the evolutionary stable allelic values ($x_{\mathrm{A}}^* \approx -1.5$ and $x_{\mathrm{a}}^* \approx 0.5$, see Fig. 1C).

With strong recombination (e.g., $r/\sigma = 0.5$), genetic values for the X and Y evolve to be distributed symmetrically around 0 (similar to autosomal chromosome pairs, Flintham et al., 2025), however, where recombination is weaker (e.g., $r/\sigma = 0.1$ or $r/\sigma = 0.05$), X-chromosome haplotypes develop that tend to encode an allelic value of 0.5 or smaller, while Y-chromosome haplotypes encode a much larger absolute effect that overshoots the male optimum in homozygous state. Indeed when linkage is tight, e.g., $r/\sigma = 0.01$, haplotype values are distributed around the evolutionary stable values (i.e., X-haplotype encoding $x_{\mathrm{a}}^* = 0.5$ and Y-linked haplotype encoding $x_{\mathrm{A}}^* = -1.5$). Consequently, when z shows a polygenic basis that is in linkage with the SDR (i.e., in the PAR), we expect that females show highest fitness when homozygous for the most common X-linked haplotype, and so are homozygotes across trait loci. Meanwhile, the highest fitness males, that is, those carrying one copy of the most common Y- and X- haplotypes, are heterozygous across loci, leading to heterozygote advantage.

C Evolution of a Y-linked suppressor

C.1 Invasion fitness

Here, we characterise the invasion fitness of a Y-linked recombination suppressor appearing as a single copy in a recruited Y-bearing sperm (i.e., that successfully goes on to form an adult male). This mutant appears arises in a population that recombines at a resident rate r and that is at a polymorphic equilibrium at the trait locus – i.e., where alleles A and a are at their equilibrium frequencies given by $\widehat{p}_{\mathrm{f}}$, $\widehat{p}_{\mathrm{m}}^{\mathrm{X}}$ and $\widehat{p}_{\mathrm{m}}^{\mathrm{Y}}$. Such a mutant arises in a Y-bearing sperm carrying allele A with probability $\widehat{p}_{\mathrm{m}}^{\mathrm{Y}}$ and one carrying allele a with probability $1-\widehat{p}_{\mathrm{m}}^{\mathrm{Y}}$. Let us denote with $W_{r,\mathrm{A}}^{\mathrm{Y}}(x_{\mathrm{a}},x_{\mathrm{A}})$ and $W_{r,\mathrm{a}}^{\mathrm{Y}}(x_{\mathrm{a}},x_{\mathrm{A}})$ the invasion fitness for each of these two cases respectively. Since we assume the suppressor fully arrests recombination with the trait locus, these are given by

$$W_{r,\mathrm{A}}^{\mathrm{Y}}(x_{\mathrm{a}},x_{\mathrm{A}}) = \frac{w_{\mathrm{m}}(x_{\mathrm{a}}+x_{\mathrm{A}})}{\overline{w}_{\mathrm{m}}}(1-\widehat{p}_{\mathrm{f}}) + \frac{w_{\mathrm{m}}(2x_{\mathrm{A}})}{\overline{w}_{\mathrm{m}}}\widehat{p}_{\mathrm{f}} \tag{C-1a}$$

$$W_{r,\mathrm{a}}^{\mathrm{Y}}(x_{\mathrm{a}},x_{\mathrm{A}}) = \frac{w_{\mathrm{m}}(2x_{\mathrm{a}})}{\overline{w}_{\mathrm{m}}}(1-\widehat{p}_{\mathrm{f}}) + \frac{w_{\mathrm{m}}(x_{\mathrm{a}}+x_{\mathrm{A}})}{\overline{w}_{\mathrm{m}}}\widehat{p}_{\mathrm{f}}, \tag{C-1b}$$

where recall $\overline{w}_{\mathrm{m}} = (1-\widehat{p}_{\mathrm{m}}^{\mathrm{Y}})(1-\widehat{p}_{\mathrm{f}})\,w_{\mathrm{m}}(2x_{\mathrm{a}}) + \widehat{p}_{\mathrm{m}}^{\mathrm{Y}}\widehat{p}_{\mathrm{f}}w_{\mathrm{m}}(2x_{\mathrm{A}}) + (\widehat{p}_{\mathrm{m}}^{\mathrm{Y}}+\widehat{p}_{\mathrm{f}}-2\widehat{p}_{\mathrm{m}}^{\mathrm{Y}}\widehat{p}_{\mathrm{f}})\,w_{\mathrm{m}}(x_{\mathrm{a}}+x_{\mathrm{A}})$ is mean male fitness with allele frequencies (see also Olito and Abbott, 2025 eq. 15 and Appendix C for an alternative derivation of an expression for a di-allelic sexually antagonistic locus with fixed effects, and Otto, 2014 for expressions for a small effect recombination modifier).

For a suppressor to invade given it arises with allele A or with allele a, we must have $W_{r,\mathrm{A}}^{\mathrm{Y}}(x_{\mathrm{a}},x_{\mathrm{A}}) > 1$ and $W_{r,\mathrm{a}}^{\mathrm{Y}}(x_{\mathrm{a}},x_{\mathrm{A}}) > 1$, respectively. We first argue that $W_{r,\mathrm{a}}^{\mathrm{Y}}(x_{\mathrm{a}},x_{\mathrm{A}}) > 1$ never holds. We do so by noting first that once alleles A and a have diverged (i.e., $x_{\mathrm{A}} < x_{\mathrm{a}}$), additive gene action entails that male heterozygotes show higher fitness than those homozygous for allele a (i.e., $w_{\mathrm{m}}(x_{\mathrm{a}}+x_{\mathrm{A}}) > w_{\mathrm{m}}(2x_{\mathrm{a}})$). Allele A can then either : (I) be strictly male-beneficial: $w_{\mathrm{m}}(2x_{\mathrm{a}}) < w_{\mathrm{m}}(x_{\mathrm{a}}+x_{\mathrm{A}}) < w_{\mathrm{m}}(2x_{\mathrm{A}})$, or (II) lead males to show heterozygote advantage: $w_{\mathrm{m}}(x_{\mathrm{a}}+x_{\mathrm{A}}) > w_{\mathrm{m}}(2x_{\mathrm{a}})$ and $w_{\mathrm{m}}(x_{\mathrm{a}}+x_{\mathrm{A}}) > w_{\mathrm{m}}(2x_{\mathrm{A}})$. In scenario (I), with some rearrangements of eq. (C-1b), it is straightforward to show that $W_{r,\mathrm{a}}^{\mathrm{Y}}(x_{\mathrm{a}},x_{\mathrm{A}}) < 1$ for all $\widehat{p}_{\mathrm{f}}$, $\widehat{p}_{\mathrm{m}}^{\mathrm{Y}}$ and all x_{a} and x_{A} (with $x_{\mathrm{A}} < x_{\mathrm{a}}$), so that a suppressor capturing allele a cannot invade. In scenario (II), rearranging eq. (C-1b) shows a suppressor can invade if the following condition holds

$$\widehat{p}_{\mathrm{f}} > \frac{w_{\mathrm{m}}(x_{\mathrm{a}}+x_{\mathrm{A}}) - w_{\mathrm{m}}(2x_{\mathrm{a}})}{2w_{\mathrm{m}}(x_{\mathrm{a}}+x_{\mathrm{A}}) - w_{\mathrm{m}}(2x_{\mathrm{a}}) - w_{\mathrm{m}}(2x_{\mathrm{A}})}. \tag{C-2}$$

This requires the equilibrium frequency of allele A be large relative to the fitness advantage of male heterozygotes. We evaluated this condition for a large range of allelic values x_a and x_A satisfying $w_m(x_a + x_A) > w_m(2x_A)$ and that would result in a balanced polymorphism at the trait-locus (Appendix A.3; including the equilibrium (x_a^*, x_A^*)), strengths of selection (σ_m and σ_f), and recombination rates r numerically (using eqs. A-9-A-10 to find the equilibrium frequencies for alleles A and a, and inserting these values into eqs. C-5b and C-2). Among 1000 parameter sets tested in $\sigma_m \in [0.0001, 1], \sigma_f \in [0.0001, 1], r \in [0.0025, 0.5], x_a \in [0.01, 0.5], x_A \in [-0.01, -1.6]$ we found none such that condition (C-2) is fulfilled.

By contrast, using the same arguments, we find that $W^Y_{r,A}(x_a, x_A) > 1$ always holds: When A is male-beneficial (scenario I above), it is straightforward to show that $W^Y_{r,A}(x_a, x_A) > 1$ is satisfied for all $\hat{p}_f, \hat{p}^Y_m, x_a$, and x_A; main text condition (C-5a). Whereas, if males show heterozygote advantage (scenario II above), then $W^Y_{r,A}(x_a, x_A) > 1$ when

$$\hat{p}_f < \frac{w_m(x_a + x_A) - w_m(2x_a)}{2w_m(x_a + x_A) - w_m(2x_a) - w_m(2x_A)} \tag{C-3}$$

(given as main text condition C-5b – eq. C-3 is the mirror image of eq. C-2). Performing the same numerical analysis as for condition eq. (C-2), we found that eq. (C-3) was fulfilled for all 1000 parameter sets we tested in $\sigma_m \in [0.0001, 1], \sigma_f \in [0.0001, 1], r \in [0.0025, 0.5], x_a \in [0.01, 0.5], x_A \in [-0.01, -1.6]$.

Having established that the capture of allele A is both a necessary and sufficient condition for invasion of a Y-linked suppressor, to simplify notation we drop the dependency of W^Y_r on allele type and use it to refer to the invasion fitness of a recombination suppressor conditional on it capturing allele A, i.e., $W^Y_r(x_a, x_A) = W^Y_{r,A}(x_a, x_A)$ (this notation is also used in the main text).

We next investigated how the strength of positive selection on a suppressor capturing allele A, measured by the magnitude of invasion fitness (i.e., how much greater than unity $W^Y_r(x_a, x_A)$ is), depends on allelic values (x_a and x_A), the strength of Gaussian sex-specific selection (σ_m and σ_f), and the recombination rate (r). As before, we used eqs. (A-9)-(A-10) to solve for $\hat{p}_f$, $\hat{p}^X_m$ and $\hat{p}^Y_m$ for a wide range of parameter sets that precipitate a balanced polymorphism at the trait locus (Appendix A.3), and plugged these frequencies and their associated parameters into eq. (C-1a) in order to produce Fig. 2. These results are summarised and interpreted in the following section (Appendix C.2).

C.2 Sexual antagonism favours recombination suppression, especially with evolutionarily stable polymorphism

In Appendix C.1 we showed that a Y-linked suppressor of recombination can invade due to selection only if it captures the male-beneficial allele A (where $x_\mathrm{A} < x^* < x_\mathrm{a}$), in which case its invasion fitness reads as

$$W_r^\mathrm{Y}(x_\mathrm{a}, x_\mathrm{A}) = \frac{w_\mathrm{m}(x_\mathrm{a}+x_\mathrm{A})(1-\widehat{p}_\mathrm{f}) + w_\mathrm{m}(2x_\mathrm{A})\widehat{p}_\mathrm{f}}{(1-\widehat{p}_\mathrm{m}^\mathrm{Y})(1-\widehat{p}_\mathrm{f})w_\mathrm{m}(2x_\mathrm{a}) + \widehat{p}_\mathrm{m}^\mathrm{Y}\widehat{p}_\mathrm{f}w_\mathrm{m}(2x_\mathrm{A}) + (\widehat{p}_\mathrm{m}^\mathrm{Y}+\widehat{p}_\mathrm{f}-2\widehat{p}_\mathrm{m}^\mathrm{Y}\widehat{p}_\mathrm{f})w_\mathrm{m}(x_\mathrm{a}+x_\mathrm{A})} \tag{C-4}$$

(eq. C-1b written out in full – although the resident recombination rate r does not appear explicitly in eq. (C-4), r does influence $W_r^\mathrm{Y}(x_\mathrm{a}, x_\mathrm{A})$ indirectly because the equilibrium frequencies of A depend on it; i.e., $\widehat{p}_\mathrm{f}$ and $\widehat{p}_\mathrm{m}^\mathrm{Y}$ depend on r, x_A and x_A). A recombination suppressor can be favoured by selection ($W_r^\mathrm{Y}(x_\mathrm{a}, x_\mathrm{A}) > 1$) via two routes: either

$$\begin{cases} w_\mathrm{m}(2x_\mathrm{A}) > w_\mathrm{m}(x_\mathrm{A}+x_\mathrm{a}), & \text{(C-5a)} \\ \text{or} & \\ \widehat{p}_\mathrm{f} < \dfrac{w_\mathrm{m}(x_\mathrm{a}+x_\mathrm{A}) - w_\mathrm{m}(2x_\mathrm{a})}{2w_\mathrm{m}(x_\mathrm{a}+x_\mathrm{A}) - w_\mathrm{m}(2x_\mathrm{a}) - w_\mathrm{m}(2x_\mathrm{A})}. & \text{(C-5b)} \end{cases}$$

The first route (eq. C-5a) is for the homozygote AA to encode the highest fitness in males (i.e. $w_\mathrm{m}(2x_\mathrm{A}) > w_\mathrm{m}(x_\mathrm{A}+x_\mathrm{a})$), whereas the second route (eq. C-5b) applies if instead the heterozygote Aa encodes the highest fitness (i.e. $w_\mathrm{m}(x_\mathrm{A}+x_\mathrm{a}) > w_\mathrm{m}(2x_\mathrm{A})$ such that males exhibit heterozygote advantage). The increased certainty in the patrilineal transmission of allele A brought by recombination suppression is advantageous if either: (a) this allele A is systematically male-beneficial such that AA homozygotes enjoy the greatest male fitness (as in condition C-5a); or (b) males show heterozygote advantage and the frequency $\widehat{p}_\mathrm{f}$ of allele A amongst eggs is sufficiently low such that a male always passing on A through his Y-chromosome produces male offspring that are mostly Aa heterozygotes (hence condition C-5b being more stringent when $\widehat{p}_\mathrm{f}$ is large).

Computing $W_r^\mathrm{Y}(x_\mathrm{a}, x_\mathrm{A})$ across a range of parameter values shows that provided a and A are maintained by balancing selection in the resident population, selection always favours a full recombination suppressor, i.e., $W_r^\mathrm{Y}(x_\mathrm{a}, x_\mathrm{A}) > 1$ (as known to be the case for a small effect recombination modifier; Otto, 2014, see her condition for selection to favour suppression with $R_m = 0$). For weakly diverged alleles (x_a and x_A similar), this is simply because condition (C-5a) is always satisfied when a and A segregate. For alleles that generate male heterozygote advantage, this tells us that under conditions leading to balancing selection on the PAR, recombination and sexually antagonistic selection always drive suf-

ficiently low frequencies $\hat{p}_{\mathrm{f}}$ of allele A in eggs to satisfy condition (C-5b).

Our results also indicate that the strength of selection on suppression is sensitive to the strength of recombination in the resident population, with $W_r^{\mathrm{Y}}(x_{\mathrm{a}}, x_{\mathrm{A}})$ being substantially greater when r is large (compare Figs. 2A-C; see also Olito and Abbott, 2025). This is because when r is small, the frequency $\hat{p}_{\mathrm{m}}^{\mathrm{Y}}$ of A in Y-bearing sperm is high anyway, which diminishes the benefit to a mutant male that ceases recombination.

Selection for recombination suppression is especially strong when alleles have evolved to their evolutionary equilibrium (i.e. $W_r^{\mathrm{Y}}(x_{\mathrm{a}}, x_{\mathrm{A}})$ is especially large when $x_{\mathrm{a}} = x_{\mathrm{a}}^*$ and $x_{\mathrm{A}} = x_{\mathrm{A}}^*$, Figs. 2A-C, white stars). This is due to two complementary effects. First, the equilibrium values x_{a}^* and x_{A}^* entail a large variance in male fitness under random mating, owing to their substantial divergence (in general, selection for recombination suppression increases with the divergence between x_{a} and x_{A}, and so is greatest under the conditions leading to male heterozygote advantage, Fig. 2). Thus, with equilibrium allelic values, Aa males experience an especially large relative fitness advantage over homozygote genotypes, which in turn leads to a strong fitness benefit to be conferred by a recombination suppressor that ensures a male's sons inherit A on their Y-chromosome. Second, the equilibrium value x_{a}^* generates the strongest possible positive selection on allele a through females (due to strong homozygote aa advantage), and so leads to the lowest equilibrium frequencies of allele A on the X-chromosome (i.e., low values of $\hat{p}_{\mathrm{f}}$ and $\hat{p}_{\mathrm{m}}^{\mathrm{X}}$). This means resident males on average carry an excess of allele a via their maternal X, and so depart more frequently from the male optimum, reinforcing the competitive advantage of males that carry allele A on their Y-chromosome.

C.3 Probability of invasion and substitution times under random mating

C.3.1 Probability of invasion

As a next step in our analysis, we calculate the probability Ψ^{Y} that a Y-linked suppressor that has arisen as a single copy successfully invades, i.e. evades stochastic loss. From Appendix C.1 and standard branching process theory, we know that a Y-linked suppressor is only under positive selection when it captures allele A; otherwise the suppressor experiences negative selection and so goes extinct with probability 1 when it arises as a single copy. The (unconditional) invasion probability of a suppressor can therefore be expressed as $\Psi^{\mathrm{Y}} = \hat{p}_{\mathrm{m}}^{\mathrm{Y}} \Psi_{\mathrm{A}}^{\mathrm{Y}}$. Here, $\hat{p}_{\mathrm{m}}^{\mathrm{Y}}$ is the frequency of allele A in Y-bearing sperm, and so gives the probability a Y-linked suppressor captures allele A, and $\Psi_{\mathrm{A}}^{\mathrm{Y}}$ is the (conditional)

probability a suppressor invades given it captured allele A.

This conditional invasion probability can be calculated using a branching process argument (Haldane, 1927; Otto and Whitlock, 2013) by solving

$$1-\Psi_{\mathrm{A}}^{\mathrm{Y}}=\sum_{j=0}^{\infty}\Pr(X=j)(1-\Psi_{\mathrm{A}}^{\mathrm{Y}})^{j}, \tag{C-6}$$

where X is a random variable denoting the number of copies produced by a rare suppressor that has captured allele A over one generation (such that $\Pr(X=j)$ for $j\in\{0,1,\ldots\}$ gives the probability mass function of suppressor fitness). Equivalently X is a random variable for the number of surviving male offspring produced by a male carrying the suppressor mutation.

Under the lifecycle given in section 2.1 (i.e. the random-union-of-gametes), X follows a Poisson distribution with mean given by $W_r^{\mathrm{Y}}(x_{\mathrm{a}},x_{\mathrm{A}})$ (shown in eq. C-1a), i.e. $X\sim\mathrm{Pois}\big(W_r^{\mathrm{Y}}(x_{\mathrm{a}},x_{\mathrm{A}})\big)$ so that

$$\Pr(X=j)=\frac{W_r^{\mathrm{Y}}(x_{\mathrm{a}},x_{\mathrm{A}})^{j}\exp\left(-W_r^{\mathrm{Y}}(x_{\mathrm{a}},x_{\mathrm{A}})\right)}{j!} \tag{C-7}$$

(we consider alternative mating system, leading to a different form for $\Pr(X=j)$ in Appendix C.3.3). Substituting eq. (C-7) into eq. (C-6), we obtain

$$1-\Psi_{\mathrm{A}}^{\mathrm{Y}}=\exp\left(-W_r^{\mathrm{Y}}(x_{\mathrm{a}},x_{\mathrm{A}})\Psi_{\mathrm{A}}^{\mathrm{Y}}\right), \tag{C-8}$$

which cannot be solved in general.

To generate our main text results, we take a numerical approach and calculate Ψ^{Y} across a wide range of allelic values x_{a} and x_{A} (including the equilibrium values x_{a}^{*} and x_{A}^{*}), strengths of Gaussian sex-specific selection σ_{m} and σ_{f}, and resident recombination rate r. This involved (1) calculating the equilibrium allele frequencies $\widehat{p}_{\mathrm{f}}$ and $\widehat{p}_{\mathrm{m}}^{\mathrm{Y}}$ and invasion fitness $W_r^{\mathrm{Y}}(x_{\mathrm{a}},x_{\mathrm{A}})$ (numerically as per Appendices A.2.1 and C.1) for a given allelic pair of values $(x_{\mathrm{a}},x_{\mathrm{A}})$ and parameter set (σ_{m},σ_{f} and r); (2) plugging $W_r^{\mathrm{Y}}(x_{\mathrm{a}},x_{\mathrm{A}})$ into eq. (C-8) and solving numerically for the conditional invasion probability $\Psi_{\mathrm{A}}^{\mathrm{Y}}$; (3) multiplying $\Psi_{\mathrm{A}}^{\mathrm{Y}}$ by $\widehat{p}_{\mathrm{m}}$ to give the unconditional probability Ψ^{Y}. Our results for Ψ^{Y} are shown in Fig. 3A and SI Fig. 4 and interpreted in main text section 3.2.

Our analysis here can be connected to that of Olito and Abbott (2025), who also calculate the unconditional invasion probability $\Psi_{\mathrm{A}}^{\mathrm{Y}}$ for a Y-linked recombination suppressor that captures a sexually antagonistic locus with fixed additive fitness effects (specifically where $w_{\mathrm{f}}(2x_{\mathrm{a}})=1$, $w_{\mathrm{f}}(x_{\mathrm{a}}+x_{\mathrm{A}})=1-s_{\mathrm{f}}/2$,

$w_f(2x_A) = 1 - s_f$, $w_m(2x_a) = 1 - s_m$, $w_m(x_a + x_A) = 1 - s_m/2$, $w_m(2x_A) = 1$ and $0 \leq s_m < 1$ and $0 \leq s_f < 1$). Olito and Abbott (2025) use eq. (C-1a) to calculate $W_r^Y(x_a, x_A)$ for a Y-suppressor capturing the strictly male-beneficial allele A and then use the approximation $\Psi_A^Y = 2s_r$ where $s_r = W_r^Y(x_a, x_A) - 1$. This works well when selection on a modifier is weak (s_r is small; e.g., under the branching process, writing $W_r^Y(x_a, x_A) = 1 + s_r$, where $s_r \ll 1$, gives $\Psi_A^Y = 2(W_r^Y(x_a, x_A) - 1) + \mathcal{O}(s_r^2)$), but strongly overestimates invasion probabilities when selection is strong (Otto and Whitlock, 2013), and so is more limited for studying recombination suppression when polymorphism evolves at PAR loci far from the SDR-boundary (r and σ large), as we do here.

C.3.2 Fixation and substitution

Our results thus far have concerned the dynamics of a suppressor as it invades into a population from a single copy. However, the tempo of recombination evolution depends not just on how frequently such suppressors invade, but also on whether they go on to fix in the population, the conditions for which may diverge from those for invasion. In particular, a large effect mutation such as a full recombination suppressor (i.e., that completely silences recombination) that invades into a population may be prevented from fixing if it experiences negative frequency-dependent selection – in which case the suppressor is held at an intermediate frequency as a balanced polymorphism.

To establish whether a Y-linked suppressor that has successfully invaded into a population will go on to fix or not, we ran individual-based simulations of a Wright-Fisher process using SLiM 4,0 (Haller and Messer, 2023) that track the frequency of a suppressor through time. Following the lifecycle in section 2.1, we simulate a diploid population of $N = 10^4$ adults with non-overlapping generations, where each individual carries a single chromosome with three loci: one XY sex-determining locus, one modifier locus that is in full linkage with the sex-determining locus, and one trait locus recombining with the sex-determining locus at rate r). At each generation, 5000 male and 5000 female individuals are created through multinomial sampling of adult males and females from the previous generation, with a sample weighting given by their fecundity ($w_m(z)$ and $w_f(z)$, which as we assume are Gaussian, where z is given by the sum of the values encoded by the alleles they carry at the trait locus). We assume the trait locus is polymorphic for alleles A and a and we initialise simulations with alleles at their equilibrium frequencies in all gene pools ($\hat{p}_m^X$, $\hat{p}_m^Y$ and $\hat{p}_f$; to ensure one allele is not lost by chance during the simulation, we allow mutations between the two alleles at a per-locus rate 5×10^{-5}).

At the beginning of each simulation, we introduce a suppressor mutant into the population, doing so in one of two different ways: (1) In one set of simulations, we introduce a single copy into the population (i.e., with initial frequency $1/(N/2)$), which is placed in the Y-chromosome of a randomly sampled male. This setup therefore follows our previous analyses in considering the fate of a novel suppressor (providing a comparison for our invasion results). (2) In a second set of simulations, we introduced the mutant into $qN/2$ (rounded to the nearest integer) randomly sampled A-bearing adult male Y-chromosomes, where $q = 1/(\Psi^{Y}_{A}N/2)$ is the frequency at which a mutant suppressor that originally arose a single copy in a chromosome containing A can be considered to have invaded (i.e., to have avoided stochastic loss when rare in a large population, Charlesworth, 2020; Maynard Smith, 1976). In other words, here we are following the trajectory of a suppressor, conditional on it successfully establishing in the population. In both cases, we ran simulations until the suppressor either fixed or went extinct in the population, doing so for the same range of parameter/allelic value combinations considered in section C.3.1, with 10^4 replicates for each parameter set.

For all parameter sets considered we found: (1) In simulations where a suppressor was introduced as a single copy, the proportion of replicates in which the mutant fixed closely matched the unconditional invasion probability, Ψ^{Y}, calculated in Appendix C.3.1 (some example values are plotted in Fig 3A, and the frequency trajectories of some example simulations are shown in Appendix Fig. 2A). (2) in simulations where the mutant was introduced at frequency q, we found that it always quickly swept to fixation. Together, these observations strongly suggest that successful invasion by a Y-suppressor is sufficient for its eventual fixation (i.e., selection on recombination suppressors is not frequency dependent even when selection is strong; this is consistent with the results of Olito and Abbott, 2025 Appendix C, and extends the weak selection results of Otto, 2014 who found that the invasion conditions for small effect recombination promoters and suppressors are mutually exclusive). Consequently, we consider that Ψ^{Y} captures both the probability of invasion and fixation for a Y-linked suppressor.

With this in mind, we can use our invasion results to calculate the expected waiting time for such a suppressor to substitute (to appear in a population and then go on to fix). To do this, we assume suppressor mutants arise in a population at a low constant rate $\lambda_{\mu} = (N/2)\mu \ll 1$ per generation (where μ is the probability a novel suppressor mutant appears in a given Y-bearing gamete), such that substitution is a Poisson process with rate $\lambda_{\text{sub}} = \lambda_{\mu}\Psi^{Y}$. From this it follows that the random variable T giving the waiting time (number of generations) for the substitution to occur is exponentially distributed

$T \sim \text{Exponential}(\lambda_{\text{sub}})$, and therefore with mean

$$\tau = \mathrm{E}[T] = 1/\lambda_{\text{sub}}. \tag{C-9}$$

To generate our main text Fig. 3B we plugged the values for Ψ^{Y} calculated numerically into eq. (C-9), which we compared against the neutral substitution time: $1/\mu$. In our figures we used a large population size 10^4 and a gametic mutation rate of 10^{-5} (as in Lenormand and Roze, 2022), although equivalent results are produced with weaker mutation rates. Recent estimates (Berdan et al., 2021, box 1) place genome-wide gametic mutation rates for recombination suppressors such as inversions at $\approx 10^{-3}$ (and chromosomal fusions at $\approx 10^{-4}$), thus our parameters are consistent with 1% of these mutations occurring on the Y-chromosome and capturing the SDR.

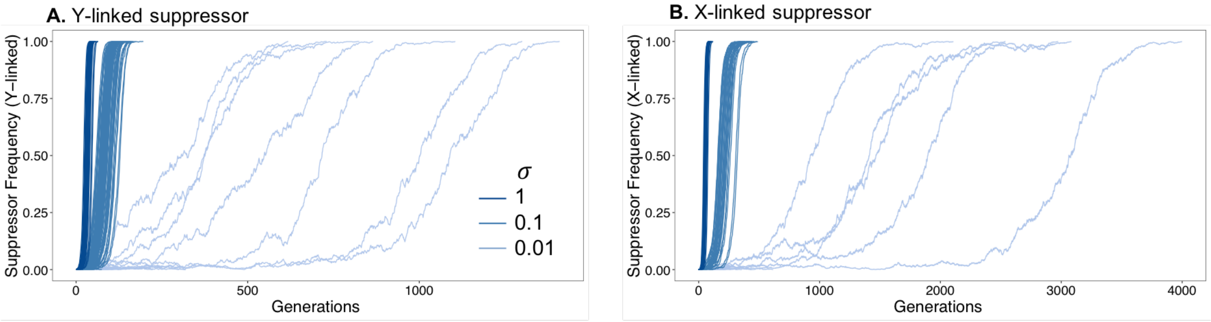

Appendix Figure 2: **Frequency of Y- and X-linked suppressors through time from example simulations**. Plots show suppressor frequency through time in example simulations where a suppressor was introduced as a single copy (Appendix C.3.2 and D.2.2 for details). Panels **A.** and **B.** respectively show frequency trajectories for simulations where a Y-linked and X-linked suppressor was introduced and different colour curves show replicates with different strengths of sexually antagonistic selection ($\sigma = \sigma_{\mathrm{f}} = \sigma_{\mathrm{m}}$, other parameters: $x_{\mathrm{a}} = 0.5, x_{\mathrm{A}} = -0.5, r = 0.5$). A sample of 1000 replicate simulations is shown for each treatment, with two mutually exclusive types of trajectories being observed for both Y- and X-linked suppressors: (1) suppressor frequency remains low after its introduction, and the mutant ends up being lost quickly, (2) suppressors rapidly sweep to fixation at a rate sensitive to σ (notice curve show the characteristic sigmoid-shape consistent with a sweep occurring). This pattern supports the notion that suppressors that successfully invade into a population systematically go on to fix.

C.3.3 Probability of invasion under polygyny

Here, we consider the invasion probability Ψ^{Y} under a model of lottery polygyny (Nunney, 1993). To do so, we can still use eq. (C-6) but X should now be non-Poisson. We determine the probability mass function $\Pr(X = j)$ under a lifecycle similar to the one analysed by Lee et al. (2008). This life-cycle is as follows : (1) $N/2$ adult males and $N/2$ adult females enter the mating pool (where N is large). Females

mate singly but males can mate multiply. For each female, a male is sampled with replacement which mates with her and sires all her offspring. The probability that a male $i \in \{1,\dots,N/2\}$ is sampled is weighted by his competitiveness which we denote as c_i (e.g., c_i represents a male's biological condition, or level of expression of a sexually selected ornament relative to other males in the population). (2) After mating, females produce a large number of juveniles (in this model, male mating success and female fecundity are independent of genotype). (3) Adults die and juveniles undergo sex-specific density-dependent competition for access to $N/2$ male and $N/2$ female adult positions in the mating pool of the next generation. This competition is mediated by the evolving trait z; i.e. the relative probability of surviving to the mating pool of a juvenile male expressing trait z is $w_{\mathrm{m}}(z)$, while that of a female is $w_{\mathrm{f}}(z)$. This ensures that the expected number of surviving offspring of males and females expressing a trait value z is identical to that in the random-union-of-gametes model (section 2.1) and therefore leads to equivalent sexually antagonistic selection in both models, allowing us to focus on the effects of mating system on reproductive variance.

For this model of polygyny, we can obtain the probability mass function $\Pr(X = j)$ for the number X of males produced by a mutant male using conditional probabilities :

$$\Pr(X = j) = \sum_{m=0}^{N/2} \sum_{\boldsymbol{f} \in \mathscr{F}} \Pr(X = j | \boldsymbol{F} = \boldsymbol{f}) \Pr(\boldsymbol{F} = \boldsymbol{f} | M = m) \Pr(M = m), \tag{C-10}$$

where M is the random variable for the number of females mated by a mutant male, and $\boldsymbol{F} = (F_{\mathrm{aa}}, F_{\mathrm{Aa}}, F_{\mathrm{AA}})$, is a vector containing the random variables for the numbers of females of each genotype the mutant male mates (e.g., F_{aa} is a random variable for the number of aa females he mates).

By definition, M has support $\{0,1,\dots,N/2\}$, and if male competitiveness follows a Beta distribution with parameters α and $\beta = \alpha(N/2-1)$ (such that $C_i \sim \mathrm{B}(\alpha, \alpha(N/2-1))$ where C_i is the random variable for competitiveness for each male i), then

$$M \sim \mathrm{BetaBin}\left(N/2, \alpha, \beta\right) \tag{C-11}$$

where BetaBin refers to the Beta-binomial distribution (Lee et al., 2008, eq. 1 with their n_i large). The random-variable M has expectation $\mathrm{E}[M] = 1$ and variance $\mathrm{Var}[M] = (N-2)(1+\alpha)/(2+N\alpha) \to 1+1/\alpha$ as $N \to \infty$. The parameter α therefore scales the variance in male mating success (through its effect on the distribution of male competitiveness), which is high when α is small and low when α is large.

Because mating is random with respect to genotype, the number of females of each genotype (AA, Aa,

and aa) that the mutant male mates with, given he mates a total m females follows a Multinomial law with parameters

$$\boldsymbol{F}|M = m \sim \text{Multinomial}(m, \boldsymbol{\pi}) \tag{C-12}$$

where $\boldsymbol{\pi} = \left((1-\widehat{p}_{\text{f}})(1-\widehat{p}^{\text{X}}_{\text{m}})\frac{w_{\text{f}}(z_{\text{aa}})}{\overline{w}_{\text{f}}}, \left(\widehat{p}_{\text{f}}(1-\widehat{p}^{\text{X}}_{\text{m}}) + (1-\widehat{p}_{\text{f}})\widehat{p}^{\text{X}}_{\text{m}}\right)\frac{w_{\text{f}}(z_{\text{Aa}})}{\overline{w}_{\text{f}}}, \widehat{p}_{\text{f}}\widehat{p}^{\text{X}}_{\text{m}}\frac{w_{\text{f}}(z_{\text{AA}})}{\overline{w}_{\text{f}}}\right)$ gives the frequencies of each genotype amongst adult females. We have denoted the support of this random variable as $\mathscr{F} = \{\boldsymbol{f} = (f_{\text{aa}}, f_{\text{Aa}}, f_{\text{AA}}) : \boldsymbol{f} \in \mathbb{Z}^3_{\geq 0}, f_{\text{aa}} + f_{\text{Aa}} + f_{\text{AA}} = m\}$.

Finally, given it mates with a group of $\boldsymbol{f} = (f_{\text{aa}}, f_{\text{Aa}}, f_{\text{AA}})$ females, the number of surviving male offspring a mutant male sires (the random variable X) is the sum of all the surviving sons of these females. According to our lifecycle (whereby females each produce a large number of juveniles that each compete independently and sex-specifically to obtain a breeding spot), the numbers of surviving male offspring of each genotype produced by a given female follow poisson distributions with means equal to the relative male fitness of each genotype weighted by the proportion of the focal female's juvenile offspring that will carry each genotype. For example, a female with genotype Aa mated to a male carrying A on his Y-chromosome will produce AA and Aa juvenile sons in equal proportions, and so will leave Poisson distributed numbers of surviving male AA and Aa offspring with means $\frac{1}{2}\frac{w_{\text{m}}(2x_{\text{A}})}{\overline{w}_{\text{m}}}$ and $\frac{1}{2}\frac{w_{\text{m}}(x_{\text{a}}+x_{\text{A}})}{\overline{w}_{\text{m}}}$, respectively. Putting all this together, we have

$$X|\boldsymbol{F} = \boldsymbol{f} \sim \text{Poisson}\left(\frac{w_{\text{m}}(x_{\text{a}} + x_{\text{A}})}{\overline{w}_{\text{m}}}(f_{\text{aa}} + f_{\text{Aa}}/2) + \frac{w_{\text{m}}(2x_{\text{A}})}{\overline{w}_{\text{m}}}(f_{\text{AA}} + f_{\text{Aa}}/2)\right). \tag{C-13}$$

Using eqs. (C-11)-(C-13) for eq. (C-10) then gives us the unconditional probability mass function $\Pr(X = j)$.

To generate main text Fig. 3C we plugged eq. (C-10) into eq. (C-6) and solved numerically for $\Psi^{\text{Y}}_{\text{A}}$, doing this for different values of α that lead to different mating variances. For computational expediency we performed the calculation with small population sizes; $N = 20$ when $\alpha \geq 1$ and $N = 100$ otherwise, but which were nonetheless sufficient to capture full probability distribution of M, i.e., $\Pr(M \leq 10) \approx 1$ when $\alpha \geq 1$ and $\Pr(M \leq 50) \approx 1$ for all other values of α considered, so that this procedure was not problematic given our broader assumption that N is large. We calculated substitution times in the same way as for the random-union-of-gametes model (described in Appendix C.3.2) to produce Fig. 3D.

In addition, we ran individual-based simulations for the fixation of a recombination suppressor under lottery polyny to compare with our numerical results for Ψ^{Y}. To apply the polygyny lifecycle, we modelled a population of fixed size N with $N/2$ male and female adults with the following stages: Each

generation, adult females independently sample a mate (i.e., males are sampled with replacement) to sire all her offspring. A males' sampling probability was weighted by his competitiveness, drawn from a Beta distribution. Females then each produce 20 juveniles. Adults die and $N/2$ juvenile males and $N/2$ juvenile females are sampled (without replacement) to become the adults of the next generation, while all other juveniles die. A male juvenile's sampling probability was weighted by $w_{\mathrm{m}}(z)$ and a female juvenile's by $w_{\mathrm{f}}(z)$. Following the same simulations procedure as in Appendix C.3.2, we computed proportion of simulations in which a novel suppressor fixed when introduced as a single copy, and found a good match with the unconditional invasion probability, Ψ^{Y}, calculated using eq. (C-10) (see dots in Fig. 3C).

D Evolution of an X-linked suppressor

D.1 Invasion fitness

In this Appendix we consider the fate of a new modifier on the X-chromosome that arises in the SDR-homologue and suppresses recombination with the trait locus in male or female heterozygotes for the modifier (as per a chromosomal inversion). Because a new X-linked suppressor may arise in either a male or a female chromosome, we compute its invasion fitness, $W^{\mathrm{X}}_{r,u}(x_{\mathrm{a}}, x_{\mathrm{A}})$, conditional on capturing allele $u \in \{\mathrm{a}, \mathrm{A}\}$, as the leading eigenvalue of the mean matrix

$$\mathrm{M}_{r,u}(x_{\mathrm{a}}, x_{\mathrm{A}}) = \begin{pmatrix} W^{\mathrm{f}\to\mathrm{f}}_{r,u}(x_{\mathrm{a}}, x_{\mathrm{A}}) & W^{\mathrm{m}\to\mathrm{f}}_{r,u}(x_{\mathrm{a}}, x_{\mathrm{A}}) \\ \\ W^{\mathrm{f}\to\mathrm{m}}_{r,u}(x_{\mathrm{a}}, x_{\mathrm{A}}) & W^{\mathrm{m}\to\mathrm{m}}_{r,u}(x_{\mathrm{a}}, x_{\mathrm{A}}) \end{pmatrix}, \tag{D-1}$$

where $W^{i\to j}_{r,u}(x_{\mathrm{a}}, x_{\mathrm{A}})$ gives the number of recruited gametes of sex $j \in \{\mathrm{f}, \mathrm{m}\}$ (i.e., number of eggs and number of sperm, respectively) containing allele $u \in \{\mathrm{a}, \mathrm{A}\}$ produced over the course of a generation

by a recruited gamete of sex $i \in \{\mathrm{f},\mathrm{m}\}$ and carrying allele $u \in \{\mathrm{a},\mathrm{A}\}$. These fitness components are:

$$
\begin{aligned}
W_{r,u}^{\mathrm{f}\to\mathrm{f}}(x_\mathrm{a},x_\mathrm{A}) =& \frac{1}{2}\left(\frac{w_\mathrm{f}(x_\mathrm{a}+x_u)}{\overline{w}_\mathrm{f}}\left(1-\widehat{p}_\mathrm{m}^\mathrm{X}\right)+\frac{w_\mathrm{f}(x_\mathrm{A}+x_u)}{\overline{w}_\mathrm{f}}\widehat{p}_\mathrm{m}^\mathrm{X}\right)\\
W_{r,u}^{\mathrm{m}\to\mathrm{f}}(x_\mathrm{a},x_\mathrm{A}) =& \frac{w_\mathrm{f}(x_\mathrm{a}+x_u)}{\overline{w}_\mathrm{f}}\left(1-\widehat{p}_\mathrm{f}\right)+\frac{w_\mathrm{f}(x_\mathrm{A}+x_u)}{\overline{w}_\mathrm{f}}\widehat{p}_\mathrm{f}\\
W_{r,u}^{\mathrm{f}\to\mathrm{m}}(x_\mathrm{a},x_\mathrm{A}) =& \frac{1}{2}\left(\frac{w_\mathrm{m}(x_\mathrm{a}+x_u)}{\overline{w}_\mathrm{m}}\left(1-\widehat{p}_\mathrm{m}^\mathrm{Y}\right)+\frac{w_\mathrm{m}(x_\mathrm{A}+x_u)}{\overline{w}_\mathrm{m}}\widehat{p}_\mathrm{m}^\mathrm{Y}\right)\\
W_{r,u}^{\mathrm{m}\to\mathrm{m}}(x_\mathrm{a},x_\mathrm{A}) =& 0.
\end{aligned}
\tag{D-2}
$$

where $\overline{w}_\mathrm{m}$ and $\overline{w}_\mathrm{f}$ are given by eq. (A-8).

The quantities $W_{r,\mathrm{A}}^\mathrm{X}(x_\mathrm{a},x_\mathrm{A})$ and $W_{r,\mathrm{a}}^\mathrm{X}(x_\mathrm{a},x_\mathrm{A})$ end up being cumbersome, and so are given in a Supplementary Mathematica Notebook. They also do not lead to readily interpretable invasion conditions ($W_{r,\mathrm{A}}^\mathrm{X}(x_\mathrm{a},x_\mathrm{A}) > 1$ and $W_{r,\mathrm{a}}^\mathrm{X}(x_\mathrm{a},x_\mathrm{A}) > 1$) for an X-linked modifier, so we again proceed with a numerical analysis. Using Gaussian functions for $w_\mathrm{f}(z)$ and $w_\mathrm{m}(z)$ and following an identical procedure as in Appendix C.1, we find that across all parameter $(\sigma_\mathrm{m},\sigma_\mathrm{f},r)$ and allelic value $(x_\mathrm{a},x_\mathrm{A})$ combinations tested, an X-linked suppressor invades only when it captures the female-beneficial allele a, and is otherwise disfavoured by selection (i.e., $W_{r,\mathrm{A}}^\mathrm{X}(x_\mathrm{a},x_\mathrm{A}) < 1$ and $W_{r,\mathrm{a}}^\mathrm{X}(x_\mathrm{a},x_\mathrm{A}) > 1$ for all $x_\mathrm{a},x_\mathrm{A},\sigma_\mathrm{m},\sigma_\mathrm{f}$ and r). We therefore drop the dependency of invasion fitness (and its components) on allele u, and use the notation $W_r^\mathrm{X}(x_\mathrm{a},x_\mathrm{A}) = W_{r,\mathrm{a}}^\mathrm{X}(x_\mathrm{a},x_\mathrm{A})$ in the main text (e.g., in section 3.3) and for the rest of the Appendix. We present plots of $W_r^\mathrm{X}(x_\mathrm{a},x_\mathrm{A})$ for different allelic values and parameters in SI Fig. 5 (calculated with the same procedure as Appendix C.1), and the strength of selection for a Y-suppressor relative to an X-suppressor ($W_r^\mathrm{Y}(x_\mathrm{a},x_\mathrm{A})/W_r^\mathrm{X}(x_\mathrm{a},x_\mathrm{A})$) in SI Fig. 6A.

D.2 Invasion and fixation probabilities for X-linked suppressors based on multitype branching process

D.2.1 Probability of invasion

As an X-linked suppressor can only be favoured by selection when it captures allele a, its unconditional probability of invasion, Ψ^X, depends on its probabilities of invasion conditional on the mutant arising on a male, $\Psi_\mathrm{m}^\mathrm{X}$, and female, $\Psi_\mathrm{f}^\mathrm{X}$, X-chromosome carrying allele a, weighted by the equilibrium frequencies of this allele; $1-\widehat{p}_\mathrm{f}$ and $1-\widehat{p}_\mathrm{m}^\mathrm{X}$. Specifically, taking into account the difference in male and female X copy numbers (and so likelihood of a given X-linked mutation first appearing in a male

or female), we have

$$\Psi^{\mathrm{X}} = \frac{2}{3}(1-\widehat{p}_{\mathrm{f}})\Psi^{\mathrm{X}}_{\mathrm{f}} + \frac{1}{3}(1-\widehat{p}^{\mathrm{X}}_{\mathrm{m}})\Psi^{\mathrm{X}}_{\mathrm{m}} \tag{D-3}$$

(Charlesworth et al., 1987).

The growth of the mutant suppressor arisen as a single copy follows a multitype branching process (Harris et al., 1963). As such, if a rare mutant can invade into a population (i.e., if $W^{\mathrm{X}}_r(x_{\mathrm{a}}, x_{\mathrm{A}}) > 1$), the probability this mutant ultimately invades conditional on it arising in a gamete produced by sex $i \in \{\mathrm{f}, \mathrm{m}\}$ is Ψ^{X}_i. If mutant fitness distributions are Poisson for all genetic backgrounds, that is, if $X^{i\to j} \sim \mathrm{Pois}\left(W^{i\to j}_{r,\mathrm{a}}(x_{\mathrm{a}}, x_{\mathrm{A}})\right)$ for all $i \in \{\mathrm{f}, \mathrm{m}\}$ and $j \in \{\mathrm{f}, \mathrm{m}\}$, where $X^{i\to j}$ is the random variable for the number of mutants in a gamete of sex j produced by a mutant in gamete of sex i, then $\mathbf{\Psi}^{\mathrm{X}} = (\Psi^{\mathrm{X}}_{\mathrm{f}}, \Psi^{\mathrm{X}}_{\mathrm{m}})$ is the unique solution to the set of simultaneous equations

$$\begin{aligned} 1-\Psi^{\mathrm{X}}_{\mathrm{f}} &= \exp\left(-W^{\mathrm{f}\to\mathrm{f}}_r(x_{\mathrm{a}}, x_{\mathrm{A}})\Psi^{\mathrm{X}}_{\mathrm{f}} - W^{\mathrm{f}\to\mathrm{m}}_r(x_{\mathrm{a}}, x_{\mathrm{A}})\Psi^{\mathrm{X}}_{\mathrm{m}}\right) \\ 1-\Psi^{\mathrm{X}}_{\mathrm{m}} &= \exp\left(-W^{\mathrm{m}\to\mathrm{f}}_r(x_{\mathrm{a}}, x_{\mathrm{A}})\Psi^{\mathrm{X}}_{\mathrm{f}} - W^{\mathrm{m}\to\mathrm{m}}_r(x_{\mathrm{a}}, x_{\mathrm{A}})\Psi^{\mathrm{X}}_{\mathrm{m}}\right) \end{aligned} \tag{D-4}$$

(where recall $W^{i\to j}_r(x_{\mathrm{a}}, x_{\mathrm{A}})$ refers to $W^{i\to j}_{r,\mathrm{a}}(x_{\mathrm{a}}, x_{\mathrm{A}})$ in eq. D-2). Taking the log of both sides of eq. (D-4) and rearranging, we arrive at the system

$$\begin{aligned} -\mathrm{Log}(1-\Psi^{\mathrm{X}}_{\mathrm{f}}) &= W^{\mathrm{f}\to\mathrm{f}}_r(x_{\mathrm{a}}, x_{\mathrm{A}})\Psi^{\mathrm{X}}_{\mathrm{f}} + W^{\mathrm{f}\to\mathrm{m}}_r(x_{\mathrm{a}}, x_{\mathrm{A}})\Psi^{\mathrm{X}}_{\mathrm{m}} \\ -\mathrm{Log}(1-\Psi^{\mathrm{X}}_{\mathrm{m}}) &= W^{\mathrm{m}\to\mathrm{f}}_r(x_{\mathrm{a}}, x_{\mathrm{A}})\Psi^{\mathrm{X}}_{\mathrm{f}} + W^{\mathrm{m}\to\mathrm{m}}_r(x_{\mathrm{a}}, x_{\mathrm{A}})\Psi^{\mathrm{X}}_{\mathrm{m}}. \end{aligned} \tag{D-5}$$

After plugging in eq. (D-2), eq. (D-5) can be solved numerically for $\Psi^{\mathrm{X}}_{\mathrm{m}}$ and $\Psi^{\mathrm{X}}_{\mathrm{f}}$ using the same approach as we used to find Ψ^{Y} in Appendix C.3.1. Finally, plugging these quantities into eq. (D-3) with the corresponding equilibrium frequencies $\widehat{p}_{\mathrm{f}}$ and $\widehat{p}^{\mathrm{X}}_{\mathrm{m}}$ allowed and comparing with the equivalent invasion probabilities for Y-linked suppressors (to calculate $\Psi^{\mathrm{Y}}/\Psi^{\mathrm{X}}$ and $\rho = \Psi^{\mathrm{Y}}/(\Psi^{\mathrm{Y}} + \Psi^{\mathrm{X}})$), we could produce Fig. 4A and SI Fig. 6B.

D.2.2 Fixation and substitution

In order to confirm that successfully invading X-linked suppressors also go on to fix, we ran individual-based simulations following the same procedure as in Appendix C.3.2. Depending on the scenario being considered, we either introduced a suppressor (1) as a single copy into the population, by placing it in a randomly sampled X-chromosome (which could be present in an adult male or female; example frequency trajectories are shown in Appendix Fig. 2B), or (2) at a frequency

$q = 1/(3N/4\Psi^X)$ by adding it to $3Nq/4$ (again rounded to the nearest integer) randomly sampled a-bearing X-chromosomes. Across the same parameter sets as used in Appendix C.3.2, we found that when introduced at frequency q, X-linked suppressors always went on to fix in the population. Furthermore, for simulations where the suppressor was introduced as a single copy, we saw a good match between the proportion of replicates in which this mutant fixed and our numerical calculation of Ψ^X in Appendix D.2.1 (see dots in Appendix Fig 3). This is consistent with invasion implying fixation for X-linked suppressors and we therefore interpret Ψ^X as both the probability of invasion and fixation for our parameter sets.

In contrast to these results, Olito and Abbott (2025) (see their Appendix C and Fig. S5) report that invading X-linked inversions capturing the SDR-homologue and the female-beneficial allele at a sexually antagonistic locus never fix (including for equivalent parameter values to those considered here), but instead reach a stable polymorphism maintained by negative frequency-dependent selection. Based on the low equilibrium frequencies they observe (typically < 0.2; Olito and Abbott, 2025, Fig. S5 C-D), the authors argue that such suppressors are likely to be lost over time and thus unlikely to contribute meaningfully to the evolution of recombination suppression. The discrepancy between our results and those of Olito and Abbott (2025) arises from two mistakes in the population genetic recursions used to find equilibrium frequencies for an inverted genotype in Olito and Abbott (2025) (Colin Olito personal communication). This paper can thus be considered an update to Olito and Abbott (2025) with respect to the substitution of X-linked inversions.

Finally, balanced polymorphisms for X-linked inversions, such that invasion does not imply fixation, have been found for some specific parameter combinations (Blackmon and Brandvain, 2017). This occurs when A and a are strictly male/female-beneficial alleles (no overshooting) and the male-beneficial allele (A) is recessive in both sexes (i.e., female-beneficial allele a dominant) (e.g., Blackmon and Brandvain, 2017, Figure 1). However such alleles are expected to contribute weakly to sexually antagonistic variation and require non additive gene action phenotype (Connallon and Chenoweth, 2019; Fry, 2010; Jordan and Charlesworth, 2012), so we do not consider them here.

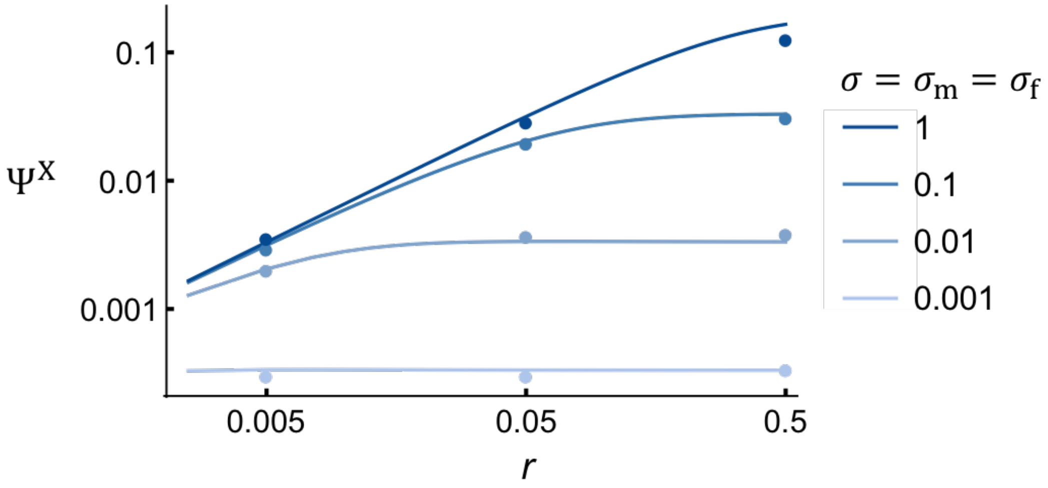

Appendix Figure 3: **Invasion of X-linked suppressors in a large population**. Figure shows the unconditional invasion probability of a newly arisen X-linked suppressor in a large population, Ψ^{X} , under the random-union-of-gametes model (Appendix D.2.1 for calculation) according to the resident recombination rate (r). Allelic values used: $x_a = 0.5$ and $x_A = -0.5$. Dots show results from individual-based simulations with (which correspond to proportion of replicate simulations in which a X-suppressor fixed, Appendix D.2.2). Different colour curves and dots correspond to results for different strengths of sexually antagonistic selection at the trait locus ($\sigma = \sigma_m = \sigma_f$).

D.3 Invasion of X-, Z- and W-suppressors under polygyny

Calculating the invasion probability of an X-linked suppressor from a multitype branching process in the presence of lottery polygyny is complex as it involves considering male and female fitness distributions (the latter of which depend on the distribution of mating success across males). We therefore estimated its fixation probability directly using individual-based simulations. This involved an identical simulation procedure as described in Appendix C.3.3, except that here a recombination suppressor was introduced as a single copy into a randomly sampled X-chromosome (rather than Y-chromosome). We then calculated the fixation probability for a given parameter set as the proportion of replicates in which the suppressor fixed. We used this proportion in place of Ψ^{X} when producing Fig. 4B.

To model the invasion of Z- and W-linked suppressors in an ZW system under polygyny, we followed a near-identical procedure as for XY simulations. The only difference here was that the SDR was now female-determining and found on the maternally inherited chromosome (W-chromosome). Suppressors then introduced on randomly sampled Z or W chromosome depending on treatment.

E Simulations of deleterious variation

In this Appendix we outline our simulation procedure for the evolution of recombination suppression in the presence of deleterious variation distributed across many loci in the PAR. Our approach follows that of Roze and Lenormand (2025) but with the addition of a sexually antagonistic locus. Specifically we consider a chromosomal region containing $L \gg 1$ deleterious loci, an SDR (modelled as a single locus), a sexually antagonistic locus, and a recombination modifier locus, giving a total of $L+3$ loci. These loci are distributed as follows: deleterious loci are found at sites $1,\ldots,L/2-1, L/2+1,\ldots,S-1, S+1,\ldots,L+2$, where S is the position of the sexually antagonistic locus, while the SDR and modifier loci are at sites $L/2$ and $L+3$, respectively. Recombination amongst adjacent sites is assumed to be uniform across the region, occurring at a rate r°. We are therefore considering a setup where an SDR is flanked by equal numbers ($L/2$) of deleterious loci to its left and right, with a sexually antagonistic locus nestled amongst the deleterious sites and whose position can be adjusted to modulate the effective recombination rate between this locus and the SDR (corresponding to the parameter r in our other models). The modifier locus occupies the terminal position and so (in the absence of a suppression mutation) does not influence recombination amongst the other loci.

During meiosis, the mean number of crossovers in our focal region is given by the map length R (unless otherwise stated, we assume $R = 1$), such that the genetic distance between adjacent sites is $R^{\circ} = R/(L+2)$. Because R is not large and we consider many deleterious loci, $R^{\circ} \ll 1$ and so closely approximates the recombination rate amongst sites, denoted r°. We therefore assume $r^{\circ} = R^{\circ}$ in our simulations. According to Haldane's mapping function (Haldane, 1919), for the sexually antagonistic locus and the SDR to recombine with probability r, they must be separated by a genetic distance $\mathrm{Log}(1/(1-2r)/2$. This entails that a number $d(r) = \mathrm{Log}(1/(1-2r))/(2R^{\circ})$ of sites sit between these two loci (we round $d(r)$ to the nearest integer), which, given the SDR is always found at position $L/2$ means that to achieve a recombination rate r we must place the sexually antagonistic locus at position $S = L/2 + d(r)$.

In the presence of deleterious variation, individuals' fitness now depends on their expression of trait z and on the alleles they carry at deleterious loci. We assume all deleterious loci are di-allelic, with a wild-type allele that does not impact fitness and a deleterious allele that reduces fecundity. Mutations is unidirectional from the wild-type to the deleterious allele at a rate $\mu_v = 10^{-4}$. The costs of deleterious alleles combine multiplicatively across loci such that a male's and female's fecundity is

given by

$$\begin{aligned} w_\mathrm{m}(z, n_1, n_2) &\propto (1-s)^{n_1}(1-hs)^{n_2} e^{-\sigma_\mathrm{m}(1+z)^2} \\ w_\mathrm{f}(z, n_1, n_2) &\propto (1-s)^{n_1}(1-hs)^{n_2} e^{-\sigma_\mathrm{f}(1-z)^2}, \end{aligned} \tag{E-1}$$

where n_1 and n_2 are the number of loci for which an individual is heterozygous and homozygous respectively, s is the homozygous selection coefficient of a deleterious allele, and h is the dominance coefficient.

We ran simulations for different parameter combinations of selection s $(0.005, 0.025, 0.125)$, dominance h $(0.1, 0.5)$ coefficients and for the numbers of deleterious loci L $(100, 500, 1000)$, and did so for an XY system, where the SDR is male-determining and paternally inherited, and a ZY system, where the SDR is female-determining and maternally inherited. For each parameter combination we simulated 10 replicate burn-in populations (in the absence of recombination modifiers and without sexually antagonistic selection: $\sigma = 0$) for 5000 generations, at which point allele frequencies at the deleterious loci had reached mutation-selection-drift equilibrium. We introduced allele A at the sexually antagonistic locus at its equilibrium frequencies in all gene pools (i.e. for XY system $\hat{p}_\mathrm{m}^\mathrm{Y}, \hat{p}_\mathrm{m}^\mathrm{X}, \hat{p}_\mathrm{f}$, see Appendix A.2.1 for calculations of these frequencies). Then, we let a single recombination suppressor arise, one that captures an entire haploid chromosome (all $L+3$ loci) at the same time. This suppressor appeared on a randomly sampled X, Y, Z, or W chromosome, depending on which type of modifier was being studied, and we tracked its frequency until it fixed or was lost. We repeated this procedure 10^6 times for each burn-in population, giving 10 estimates of the fixation probability for each parameter combination. Where relevant, all other aspects of the simulation proceed as in Appendix C.3.2.

References

Andersson, M. B. (1994). *Sexual selection.* Princeton University Press.

Bachtrog, D. (2013). Y-chromosome evolution: emerging insights into processes of Y-chromosome degeneration. *Nature Reviews Genetics*, 14(2):113–124.

Bachtrog, D., Mank, J. E., Peichel, C. L., Kirkpatrick, M., Otto, S. P., Ashman, T.-L., Hahn, M. W., Kitano, J., Mayrose, I., Ming, R., et al. (2014). Sex determination: why so many ways of doing it? *PLoS biology*, 12(7):e1001899.

Berdan, E. L., Barton, N. H., Butlin, R., Charlesworth, B., Faria, R., Fragata, I., Gilbert, K. J., Jay, P., Kapun, M., Lotterhos, K. E., et al. (2023). How chromosomal inversions reorient the evolutionary process. *Journal of Evolutionary Biology*, 36(12):1761–1782.

Berdan, E. L., Blanckaert, A., Slotte, T., Suh, A., Westram, A. M., and Fragata, I. (2021). Unboxing mutations: Connecting mutation types with evolutionary consequences. *Molecular Ecology*, 30(12):2710–2723.

Bergero, R. and Charlesworth, D. (2009). The evolution of restricted recombination in sex chromosomes. *Trends in ecology & evolution*, 24(2):94–102.

Beukeboom, L. W. and Perrin, N. (2014). *The evolution of sex determination.* Oxford University Press.

Blackmon, H. and Brandvain, Y. (2017). Long-term fragility of Y chromosomes is dominated by short-term resolution of sexual antagonism. *Genetics*, 207(4):1621–1629.

Blaser, O., Grossen, C., Neuenschwander, S., and Perrin, N. (2013). Sex-chromosome turnovers induced by deleterious mutation load. *Evolution*, 67(3):635–645.

Blaser, O., Neuenschwander, S., and Perrin, N. (2014). Sex-chromosome turnovers: the hot-potato model. *The American Naturalist*, 183(1):140–146.

Brown, J. S. and Vincent, T. L. (1987). A theory for the evolutionary game. *Theoretical Population Biology*, 31(1):140–166.

Charlesworth, B. (2020). How long does it take to fix a favorable mutation, and why should we care? *The American Naturalist*, 195(5):753–771.

Charlesworth, B., Coyne, J. A., and Barton, N. H. (1987). The relative rates of evolution of sex chromosomes and autosomes. *The American Naturalist*, 130(1):113–146.

Charlesworth, B. and Olito, C. (2024). Making sense of recent models of the "sheltering" hypothesis for recombination arrest between sex chromosomes. *Evolution*, page qpae119.

Charlesworth, D. (2017). Evolution of recombination rates between sex chromosomes. *Philosophical Transactions of the Royal Society B: Biological Sciences*, 372(1736):20160456.

Charlesworth, D. and Charlesworth, B. (1980). Sex differences in fitness and selection for centric fusions between sex-chromosomes and autosomes. *Genetics Research*, 35(2):205–214.

Charlesworth, D. and Charlesworth, B. (2005). Steps in the evolution of heteromorphic sex chromosomes. *Heredity*, 95(2):118–128.

Clark, A. (1988). The evolution of the Y chromosome with XY recombination. *Genetics*, 119(3):711–720.

Clutton-Brock, T. H. and Parker, G. A. (1992). Potential reproductive rates and the operation of sexual selection. *The Quarterly Review of Biology*, 67(4):437–456.

Connallon, T. and Chenoweth, S. F. (2019). Dominance reversals and the maintenance of genetic variation for fitness. *PLoS biology*, 17(1):e3000118.

Connallon, T. and Clark, A. G. (2014a). Balancing selection in species with separate sexes: insights from fisher's geometric model. *Genetics*, 197(3):991–1006.

Connallon, T. and Clark, A. G. (2014b). Evolutionary inevitability of sexual antagonism. *Proceedings of the Royal Society B: Biological Sciences*, 281(1776):20132123.

Dercole, F. and Rinaldi, S. (2008). *Analysis of evolutionary processes.* Princeton University Press.

Fisher, R. A. (1931). The evolution of dominance. *Biological Reviews of the Cambridge Philosophical Society*, 6(4):345–368.

Fisher, R. A. (1958). *The genetical theory of natural selection.* Oxford University Press.

Flintham, E., Savolainen, V., Otto, S. P., Reuter, M., and Mullon, C. (2025). The maintenance of genetic polymorphism underlying sexually antagonistic traits. *Evolution letters*, 9(2):259–272.

Fry, J. D. (2010). The genomic location of sexually antagonistic variation: some cautionary comments. *Evolution*, 64(5):1510–1516.

Gavrilets, S. and Rice, W. R. (2006). Genetic models of homosexuality: generating testable predictions. *Proceedings of the Royal Society B: Biological Sciences*, 273(1605):3031–3038.

Geritz, S. A., Kisdi, E., Metz, J. A., et al. (1998). Evolutionarily singular strategies and the adaptive growth and branching of the evolutionary tree. *Evolutionary ecology*, 12(1):35–57.

Haldane, J. B. (1919). The combination of linkage values, and the calculation of distances between the loci of linked factors. *Journal of Genetics*, 8:299–309.

Haldane, J. B. S. (1927). A mathematical theory of natural and artificial selection, part v: selection and mutation. In *Mathematical Proceedings of the Cambridge Philosophical Society*, volume 23, pages 838–844. Cambridge University Press.

Haller, B. C. and Messer, P. W. (2023). Slim 4: multispecies eco-evolutionary modeling. *The American Naturalist*, 201(5):E127–E139.

Harris, T. E. et al. (1963). *The theory of branching processes*, volume 6. Springer Berlin.

Ironside, J. E. (2010). No amicable divorce? Challenging the notion that sexual antagonism drives sex chromosome evolution. *BioEssays*, 32(8):718–726.

Janicke, T., Häderer, I. K., Lajeunesse, M. J., and Anthes, N. (2016). Darwinian sex roles confirmed across the animal kingdom. *Science advances*, 2(2):e1500983.

Jay, P., Jeffries, D., Hartmann, F. E., Véber, A., and Giraud, T. (2024). Why do sex chromosomes progressively lose recombination? *Trends in Genetics*.

Jeffries, D., Benvenuto, C., Böhne, A., Fraïsse, C., Garcia, S., Jay, P., Kratochvíl, L., McDonough-Goldstein, C. E., Ruiz-Herrera, A., Sotero-Caio, C. G., et al. (2025). The tree of sex consortium: A global initiative for studying the evolution of reproduction in eukaryotes. *Journal of evolutionary biology*, 38(7):861–886.

Jeffries, D. L., Gerchen, J. F., Scharmann, M., and Pannell, J. R. (2021). A neutral model for the loss of recombination on sex chromosomes. *Philosophical Transactions of the Royal Society B: Biological Sciences*, 376(1832):20200096.

Jordan, C. Y. and Charlesworth, D. (2012). The potential for sexually antagonistic polymorphism in different genome regions. *Evolution*, 66(2):505–516.

Kidwell, J., Clegg, M., Stewart, F., and Prout, T. (1977). Regions of stable equilibria for models of differential selection in the two sexes under random mating. *Genetics*, 85(1):171–183.

Kimura, M. (1965). A stochastic model concerning the maintenance of genetic variability in quantitative characters. *Proceedings of the National Academy of Sciences*, 54(3):731–736.

Kirkpatrick, M. (2010). How and why chromosome inversions evolve. *PLoS biology*, 8(9):e1000501.

Lande, R. (1980). Sexual dimorphism, sexual selection, and adaptation in polygenic characters. *Evolution*, 34(2):292–305.

Laporte, V. and Charlesworth, B. (2002). Effective population size and population subdivision in demographically structured populations. *Genetics*, 162(1):501–519.

Lee, A. M., Engen, S., and Sæther, B.-E. (2008). Understanding mating systems: a mathematical model of the pair formation process. *Theoretical Population Biology*, 73(1):112–124.

Lehtonen, J. (2022). Bateman gradients from first principles. *Nature Communications*, 13(1):3591.

Lenormand, T. (2003). The evolution of sex dimorphism in recombination. *Genetics*, 163(2):811–822.

Lenormand, T. and Roze, D. (2022). Y recombination arrest and degeneration in the absence of sexual dimorphism. *Science*, 375(6581):663–666.

Lenormand, T. and Roze, D. (2024). Can mechanistic constraints on recombination reestablishment explain the long-term maintenance of degenerate sex chromosomes? *Peer Community Journal*, 4.

Liu, Z., Herbert, A. L., Chan, Y. F., Kučka, M., Kingsley, D. M., and Peichel, C. L. (2025). The fourspine stickleback (*Apeltes quadracus*) has an XY sex chromosome system with polymorphic inversions on both X and Y chromosomes. *PLoS genetics*, 21(5):e1011465.

Ma, W.-J. and Rovatsos, M. (2022). Sex chromosome evolution: The remarkable diversity in the evolutionary rates and mechanisms. *Journal of Evolutionary biology*, 35(12):1581–1588.

Maynard Smith, J. (1976). What determines the rate of evolution? *The American Naturalist*, 110(973):331–338.

McAllister, B. F. (2003). Sequence differentiation associated with an inversion on the neo-x chromosome of drosophila americana. *Genetics*, 165(3):1317–1328.

Moraga, C., Branco, C., Rougemont, Q., Jedlička, P., Mendoza-Galindo, E., Veltsos, P., Hanique, M., de la Vega, R. C. R., Tannier, E., Liu, X., Lemaitre, C., Fields, P. D., Cruaud, C., Labadie, K., Belser, C., Briolay, J., Santoni, S., Cegan, R., Linheiro, R., Adam, G., Filali, A. E., Mossion, V., Boualem,

A., Tavares, R., Chebbi, A., Cordaux, R., Fruchard, C., Prentout, D., Velt, A., Spataro, B., Delmotte, S., Weingartner, L., Toegelová, H., Tulpová, Z., Cápal, P., Šimková, H., Štorchová, H., Krüger, M., Abeyawardana, O. A. J., Taylor, D. R., Olson, M. S., Sloan, D. B., Karrenberg, S., Delph, L. F., Charlesworth, D., Muyle, A., Giraud, T., Bendahmane, A., Genova, A. D., Madoui, M.-A., Hobza, R., and Marais, G. A. B. (2025). The *Silene latifolia* genome and its giant Y chromosome. *Science*, 387(6734):630–636.

Muralidhar, P. and Coop, G. (2024). Polygenic response of sex chromosomes to sexual antagonism. *Evolution*, 78(3):539–554.

Nei, M. (1969). Linkage modification and sex difference in recombination. *Genetics*, 63(3):681.

Nunney, L. (1993). The influence of mating system and overlapping generations on effective population size. *Evolution*, 47(5):1329–1341.

Olito, C. and Abbott, J. K. (2025). The evolution of suppressed recombination between sex chromosomes and the lengths of evolutionary strata. *Evolution*, 79(7):1371–1385.

Olito, C., Ponnikas, S., Hansson, B., and Abbott, J. K. (2024). Consequences of partially recessive deleterious genetic variation for the evolution of inversions suppressing recombination between sex chromosomes. *Evolution*, 78(8):1499–1510.

Otto, S. P. (2014). Selective maintenance of recombination between the sex chromosomes. *Journal of Evolutionary Biology*, 27(7):1431–1442.

Otto, S. P. and Day, T. (2011). *A Biologist's Guide to Mathematical Modeling in Ecology and Evolution*. Princeton University Press.

Otto, S. P., Pannell, J. R., Peichel, C. L., Ashman, T.-L., Charlesworth, D., Chippindale, A. K., Delph, L. F., Guerrero, R. F., Scarpino, S. V., and McAllister, B. F. (2011). About PAR: the distinct evolutionary dynamics of the pseudoautosomal region. *Trends in Genetics*, 27(9):358–367.

Otto, S. P. and Whitlock, M. C. (2013). *Fixation Probabilities and Times*. John Wiley & Sons, Ltd.

Pokorna, M. and Kratochvíl, L. (2009). Phylogeny of sex-determining mechanisms in squamate reptiles: are sex chromosomes an evolutionary trap? *Zoological Journal of the Linnean Society*, 156(1):168–183.

Ponnikas, S., Sigeman, H., Abbott, J. K., and Hansson, B. (2018). Why do sex chromosomes stop recombining? *Trends in Genetics*, 34(7):492–503.

Roze, D. and Lenormand, T. (2025). Effects of deleterious mutations on the fixation of chromosomal inversions on autosomes and sex chromosomes. *bioRxiv*.

Rueffler, C., Van Dooren, T. J., Leimar, O., and Abrams, P. A. (2006). Disruptive selection and then what? *Trends in Ecology & Evolution*, 21(5):238–245.

Shuker, D. M. and Simmons, L. W. (2014). *The evolution of insect mating systems*. Number 27. Oxford University Press, USA.

Vicoso, B. (2019). Molecular and evolutionary dynamics of animal sex-chromosome turnover. *Nature ecology & evolution*, 3(12):1632–1641.

Vicoso, B. and Charlesworth, B. (2009). Effective population size and the faster-x effect: an extended model. *Evolution*, 63(9):2413–2426.

Wade, M. J. and Shuster, S. M. (2004). Sexual selection: harem size and the variance in male reproductive success. *The American Naturalist*, 164(4):E83–E89.

Wilson, A. J. and Nussey, D. H. (2010). What is individual quality? An evolutionary perspective. *Trends in ecology & evolution*, 25(4):207–214.

Wright, A. E., Dean, R., Zimmer, F., and Mank, J. E. (2016). How to make a sex chromosome. *Nature communications*, 7(1):12087.

Yue, J., Krasovec, M., Kazama, Y., Zhang, X., Xie, W., Zhang, S., Xu, X., Kan, B., Ming, R., and Filatov, D. A. (2023). The origin and evolution of sex chromosomes, revealed by sequencing of the *Silene latifolia* female genome. *Current Biology*, 33(12):2504–2514.

Zhou, Q., Zhang, J., Bachtrog, D., An, N., Huang, Q., Jarvis, E. D., Gilbert, M. T. P., and Zhang, G. (2014). Complex evolutionary trajectories of sex chromosomes across bird taxa. *Science*, 346(6215):1246338.
